# Quality indicators in intensive care medicine for Germany – fourth edition 2022

**DOI:** 10.3205/000324

**Published:** 2023-06-23

**Authors:** Oliver Kumpf, Markus Assenheimer, Frank Bloos, Maria Brauchle, Jan-Peter Braun, Alexander Brinkmann, Patrick Czorlich, Christof Dame, Rolf Dubb, Georg Gahn, Clemens-A. Greim, Bernd Gruber, Hilmar Habermehl, Egbert Herting, Arnold Kaltwasser, Sabine Krotsetis, Bastian Kruger, Andreas Markewitz, Gernot Marx, Elke Muhl, Peter Nydahl, Sabrina Pelz, Michael Sasse, Stefan J. Schaller, Andreas Schäfer, Tobias Schürholz, Marina Ufelmann, Christian Waydhas, Jörg Weimann, René Wildenauer, Gabriele Wöbker, Hermann Wrigge, Reimer Riessen

**Affiliations:** 1 Charité – Universitätsmedizin Berlin, corporate member of Freie Universität Berlin and Humboldt-Universität zu Berlin, Department of Anesthesiology and Intensive Care Medicine, Berlin, Germany; 2 Diakoneo Diak Klinikum Schwäbisch Hall, Schwäbisch Hall, Germany; 3 Jena University Hospital, Department of Anaesthesiology and Intensive Care Medicine, Jena, Germany; 4 Landeskrankenhaus Feldkirch, Department of Anesthesiology and Intensive Care Medicine, Feldkirch, Austria; 5 Martin-Luther-Krankenhaus, Department of Anesthesiology and Intensive Care Medicine, Berlin, Germany; 6 Klinikum Heidenheim, Department of Anesthesia, Surgical Intensive Care Medicine and Special Pain Therapy, Heidenheim, Germany; 7 University Medical Center Hamburg-Eppendorf, Department of Neurosurgery, Hamburg, Germany; 8 Charité – Universitätsmedizin Berlin, corporate member of Freie Universität Berlin and Humboldt-Universität zu Berlin, Department of Neonatology, Berlin, Germany; 9 Kreiskliniken Reutlingen, Academy of the District Hospitals Reutlingen, Germany; 10 Städt. Klinikum Karlsruhe gGmbH, Department of Neurology, Karlsruhe, Germany; 11 Klinikum Fulda, Department of Anesthesia and Surgical Intensive Care Medicine, Fulda, Germany; 12 Niels Stensen Clinics, Marienhospital Osnabrueck, Department Hospital Hygiene, Osnabrueck, Germany; 13 Kreiskliniken Reutlingen, Klinikum am Steinenberg, Center for Intensive Care Medicine, Reutlingen, Germany; 14 Universitätsklinikum Schleswig-Holstein, Department of Pediatrics and Adolescent Medicine, Campus Lübeck, Germany; 15 Universitätsklinikum Schleswig-Holstein, Nursing Development and Nursing Science, affiliated with the Nursing Directorate Campus Lübeck, Germany; 16 Bendorf, Germany; 17 University Hospital RWTH Aachen, Department of Intensive Care Medicine and Intermediate Care, Aachen, Germany; 18 Groß-Grönau, Germany; 19 Universitätsklinikum Schleswig-Holstein, Nursing Development and Nursing Science, affiliated with the Nursing Directorate Campus Kiel, Germany; 20 Universitäts- und Rehabilitationskliniken Ulm, Intensive Care Unit, Ulm, Germany; 21 Medizinische Hochschule Hannover, Department of Pediatric Cardiology and Pediatric Intensive Care Medicine, Hanover, Germany; 22 Technical University of Munich, School of Medicine, Klinikum rechts der Isar, Department of Anesthesiology and Intensive Care Medicine, Munich, Germany; 23 Klinikum Kassel, Germany; 24 Technical University of Munich, Klinikum rechts der Isar, Department of Nursing, Munich, Germany; 25 Berufsgenossenschaftliches Universitätsklinikum Bergmannsheil, Surgical University Hospital and Polyclinic, Bochum, Germany; 26 Medical Department of the University of Duisburg-Essen, Essen, Germany; 27 Sankt-Gertrauden Krankenhaus, Department of Anesthesia and Interdisciplinary Intensive Care Medicine, Berlin, Germany; 28 Hausarztzentrum Wiesentheid, Germany; 29 Helios Universitätsklinikum Wuppertal, Universität Witten-Herdecke, Department of Intensive Care Medicine, Wuppertal, Germany; 30 Bergmannstrost Hospital Halle, Department of Anesthesiology, Intensive Care and Emergency Medicine, Pain Therapy, Halle, Germany; 31 Martin-Luther University Halle-Wittenberg, Medical Faculty, Halle, Germany; 32 Universitätsklinikum Tübingen, Department of Internal Medicine, Medical Intensive Care Unit, Tübingen, Germany

**Keywords:** quality management, intensive care medicine, quality indicators, peer review

## Abstract

The measurement of quality indicators supports quality improvement initiatives. The German Interdisciplinary Society of Intensive Care Medicine (DIVI) has published quality indicators for intensive care medicine for the fourth time now. After a scheduled evaluation after three years, changes in several indicators were made. Other indicators were not changed or only minimally. The focus remained strongly on relevant treatment processes like management of analgesia and sedation, mechanical ventilation and weaning, and infections in the ICU. Another focus was communication inside the ICU. The number of 10 indicators remained the same. The development method was more structured and transparency was increased by adding new features like evidence levels or author contribution and potential conflicts of interest. These quality indicators should be used in the peer review in intensive care, a method endorsed by the DIVI. Other forms of measurement and evaluation are also reasonable, for example in quality management. This fourth edition of the quality indicators will be updated in the future to reflect the recently published recommendations on the structure of intensive care units by the DIVI.

## Abstract (de)

Die Messung relevanter Qualitätsindikatoren unterstützt Initiativen zur Qualitätsverbesserung. Die Deutsche Interdisziplinäre Vereinigung für Intensiv- und Notfallmedizin (DIVI) hat die intensivmedizinischen Qualitätsindikatoren nun zum vierten Mal publiziert. Sie wurden nach drei Jahren überarbeitet und einige Indikatoren wurden angepasst. Andere Indikatoren erfuhren keine oder nur minimale Änderungen. Der Fokus besteht weiter auf relevanten Behandlungsprozessen wie Management von Analgesie und Sedierung, Beatmung und Weaning sowie Infektionen auf der Intensivstation. Die Gesamtzahl von zehn Indikatoren blieb bestehen. Die Entwicklung der Indikatoren erfolgte in der neuen Version nach einem anderen Prozess. Die Transparenz wurde durch Erwähnung der Einzelbeiträge der Autoren sowie auch potenzieller Interessenkonflikte erhöht. Die Anwendung der Qualitätsindikatoren im Peer-Review-Prozess der DIVI ist weiter wesentlicher Schwerpunkt. Aber auch andere Anwendungen, z.B. im Qualitätsmanagement, sind sinnvoll. Diese vierte Auflage der Qualitätsindikatoren wird noch einmal aktualisiert, um die kürzlich veröffentlichten Empfehlungen der DIVI zur Struktur der Intensivstationen zu berücksichtigen.

## Introduction

Since 2010 the German Interdisciplinary Association for Intensive Care and Emergency Medicine (DIVI) has been publishing Quality Indicators (QIs) for intensive care in Germany [1]. Following updates in 2013 [2] and 2017 [3], the fourth edition has now been completed. These QIs are an integral part of the peer review process in intensive care, which is based on recommendations by the DIVI [4], [5]. The development of this version included additional steps to improve the QIs and to substantiate their scientific basis. Methodologically the QIs remain mostly process indicators. They are focused on the evaluation of frequently performed medical processes in intensive care units (ICUs) with high relevance for treatment outcome.

Experience from external peer reviews in ICUs shows that many established processes still need improvement [6]. Compared to other quality-relevant topics, such as staffing and organization, QIs are often regarded as less important and are, therefore, not consistently and broadly applied and reported [4]. QIs are also in part revenue-relevant, as they are used to determine structural features of ICUs. They are therefore also important at a political level and serve in part as criteria for external quality assurance. However, there is also a risk of misuse if QIs are used for revenue-relevant or economic purposes.

Primary use of QIs should be the evaluation of relevant treatment processes in intensive care and the initiation of an improvement process. They are intended to support the improvement of medical and nursing quality. The indicators are developed by interprofessional and interdisciplinary working groups to evaluate actual and targeted quality levels. However, the goal is a continuous quality improvement and the adaption of the targets rather than reaching the minimum requirements of QI targets.

## Development of the fourth version of the quality indicators for intensive care

The National Steering Group for Peer Review in Intensive Care (NSPR) of the DIVI develops the QIs by employing current guidelines and new evidence from the literature on a regular basis [7]. During the current development, the QUALIFY instrument was used for the first time to evaluate the previous version of the QI [8], [9]. In a qualitative survey, the members of the steering group evaluated the relevance and applicability of the QIs, and aspects such as scientific justification and potential undesirable effects were considered as well. This provided valuable information for the further development of the QIs. Overall, it seemed that the QIs complied with the requirements described in the QUALIFY instrument. The formal structure of the QIs was modified, including the report of evidence levels and the definition of the relevant patient population. The overall number of 10 indicators remained the same and was regarded as useful and manageable. In addition, the evaluation revealed that the thematic focus of the QIs should also remain unchanged. The format of the tabular presentation was adapted to include potential conflicts of interest of the authors.

The revision started in March 2020. The medical societies organized in the DIVI which are also represented in the NSPR and other stakeholders societies discussed the priorities after an indepth evaluation of the existing QIs according to the process described above. In several Delphi rounds, the topics of the QIs were defined. Further development in individual working groups (WG) followed. After consensus within the NSPR, the Executive Board (Presidium) of the DIVI confirmed the quality indicators for intensive care in March 2022 and granted the approval for publication.

## Comparison of intensive care quality indicators

DIVI quality indicators focus on process analysis [5], [10]. In contrast, most other countries and the European Society of Intensive Care Medicine (ESICM) use outcome indicators [11], [12]. However, in our opinion processes of intensive care have the greatest influence on the actual outcome of a particular treatment. A disadvantage of these process indicators is the need for regular updates [7], [13]. The QIs of the DIVI should be seen in a broader context of measures for quality assurance. Another advantage of process based quality indicators is that implementation is not linked to profound structural changes.

## Application of intensive care quality indicators

Intensive care medicine should be based on agreed guidelines and recommendations. Therefore, the implementation of guideline-based treatment processes is the main objective of QIs. Unfortunately, there are currently no data on widespread and regular evaluation of QIs in Germany except for observations in intensive care peer reviews [4]. A key objective in the coming years is to create a better database for this purpose. The broad application of electronic patient data-management systems (PDMS) and regular data collection regarding QIs is a way to achieve this goal.

**Figure 1:**
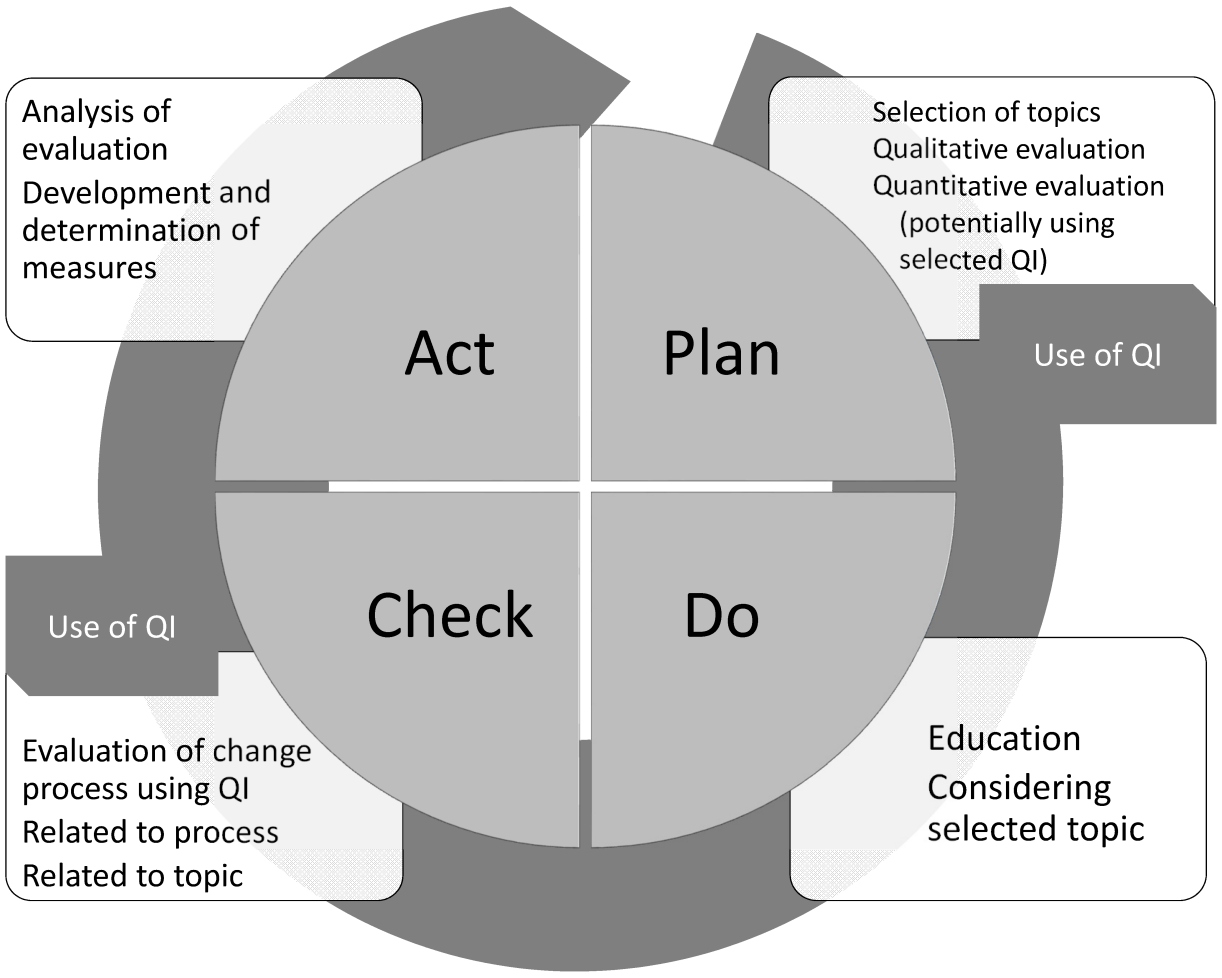
Introduction of quality indicators using the PDCA cycle (Plan=P; D=Do; C=Check; A=Act). QI=quality indicator. Use of quality indicators in intensive care considering the PDCA cycle: QI can be used to support planning in recording an actual state. The main benefit is to check the effectiveness of the measures introduced in terms of a link between “check” and “act”.

Voluntary peer reviews as recommended and supported by the DIVI may also be an important way to gather data on QIs. Ideally, all of the QIs are evaluated completely and on a regular basis. They then can be integrated in an internal quality management system (see Figure 1 (Fig. 1)). To date this is not possible everywhere for various reasons. For example, PDMS, which would facilitate quality management measures by providing data for indicators, are not broadly available in German ICUs. Moreover, existing systems do not offer integrated functions for quality evaluation [14]. Data protection issues also need to be addressed in this context.

## The future development of the DIVI quality indicators

The DIVI QIs have certainly gained relevance in recent years, and their use is not limited to the medical processes described. In part, elements of the QIs will further be used to define structural requirements and are used for other purposes than peer review, e.g. external quality assurance. They are also used by health care insurers to derive rules for reimbursement of patients treated in an ICU. Since the QIs were not designed or evaluated for this purpose, this might be not adequate or justified. Therefore, the generation of data on the effectiveness of the QI application will be an essential part in the future development of QIs [8]. The implementation and use of QIs should be continuously improved using relevant evidence from the literature. Broad application of QIs, data collection and evaluation will be of considerable importance to assess their efficacy. Finally, we believe quality evaluation in intensive care through the use of QIs will also impact reimbursement schemes.

This new version of the QIs will be an integral part of the peer review process in intensive care advocated by the DIVI. This external peer review will continue to be a very important element of quality assurance in intensive care medicine. Therefore, the development of QIs will remain one of the DIVI’s key activities.

## The fourth edition of the intensive care quality indicators for Germany

Similar to the previous publications of the quality indicators, each one is explained in terms of its underlying evidence. The list of the QIs is shown in tabular form in Attachment 1. The tabular form of the QIs, which includes author contributions and potential conflicts of interest, was published on March 14, 2022, on the DIVI’s website (https://www.divi.de/empfehlungen/qualitaetssicherung-intensivmedizin/peer-review/qualitaetsindikatoren).

### QI I Daily multi-professional and interdisciplinary ward rounds with documentation of daily goals

(Working group (WG): R. Wildenauer, A. Brinkmann, A. Markewitz, M. Assenheimer)

The daily ICU round is an integrating, communication-promoting and outcome-relevant component of the care of critically ill patients. It provides guidance for the interdisciplinary team through the definition of daily goals. Due to this interdisciplinary and interprofessional communication, the (at least) daily updated treatment plan can be discussed and demonstrably improved [15].

The medical and nursing team should consist at least of the responsible senior physician (responsible for all decisions), junior physicians, and the responsible nurse, as well as the ward manager. Other professions, such as physiotherapy, speech therapy, microbiology, clinical pharmacy, or psychology, may be involved as well; as far as possible the critically ill patient should be integrated into this round.

The use of acronyms (see tabular form in Attachment 1) for the evaluation of daily goals of treatment with regard to ethical aspects and for reviewing indications of therapies facilitates a focused and integrative approach, especially if involved disciplines cannot take part simultaneously [16], [17]. However, the use of a checklist only could neglect a more holistic view of patient care.

Ideally an electronic documentation system (PDMS) supports the comprehensibility of daily therapy goals as well as the evaluation of the quality indicator in the context of an external peer review. In addition to this aspect, relevant data for reimbursement purposes are easier to collect electronically, too. Therefore, further implementation of electronic patient records is necessary and should be promoted [14].

### QI II Management of sedation, analgesia, and delirium

(WG: P. Czorlich, O. Kumpf, S. Krotsetis)

Inadequate sedation (oversedation or undersedation), inadequate analgesia and untreated delirium result in increased morbidity, mortality, and resource use. The aim of this indicator is to avoid sedation and to reduce its duration in all critically ill patients, in addition to the adequate diagnosis and treatment of delirium. The German S3-guideline published in 2021 is the scientific basis for the indicator [18]. The mathematical formula includes all three aspects: delirium, analgesia, and sedation. The indicator includes the implementation of a guideline-based, multimodal concept (i.e. standard of care) for managing analgesia, sedation, and delirium in each ICU. Process implementation is monitored by measuring sedation depth, analgesia quality, and signs of delirium at least every eight hours. The reference value of the indicator has been changed. It allows only one missing value per 24 hours in the new version. The evaluation of an outcome indicator is recommended as well.

### QI III Patient-adapted ventilation (for severe pulmonary failure)

(WG: H. Wrigge, O. Kumpf, P. Schürholz, B. Kruger)

The topic of this indicator remained unchanged and aims to improve treatment outcomes for patients with severe pulmonary failure. The focus is patient-adapted ventilation to ensure adequate gas exchange while avoiding secondary lung damage. A protective ventilation strategy is particularly important in this regard. In daily clinical practice this is still not consistently implemented in many ICUs [19]. A guideline-based treatment standard for patient-adapted mechanical ventilation should be established in every ICU. This should include tidal volume limitation (V_T_ 6–7 ml/kg ideal body weight), plateau pressure limitation (<30 cmH_2_O), and individualized positive end-expiratory pressure (PEEP) setting. Driving pressure (plateau pressure minus PEEP or V_T_ divided by compliance of the respiratory system) takes not only V_T_ into account but also compliance. Thus, it is better suited for estimating the individual mechanical strain of ventilation than V_T_ alone, as has been shown by a strong association with survival in patients with acute respiratory distress syndrome (ARDS) [19], [20]. Individualization of PEEP can be achieved according to the extent of hypoxemia present (e.g. using a PEEP-table). In patients with severely compromised oxygenation, i.e. PaO_2_/F_I_O_2_<150 mmHg, prone positioning (180°) is recommended. Use of muscle relaxants is not recommended, even in the early stages of ARDS [21]. In the case of most severe pulmonary failure, cooperation with a specialized center for extracorporeal lung support is recommended [22].

### QI IV Early weaning from invasive ventilation

(WG: H. Habermehl, O. Kumpf, R. Riessen)

The timely and successful weaning from invasive ventilation is one of the most important goals in intensive care in order to avoid ventilation-associated complications. Early spontaneous breathing trials (SBT) help to maintain muscle function and contribute to individual rehabilitation. The aim is to keep the duration of ICU treatment as short as possible, especially in times of limited capacities. Additionally, the number of patients admitted to home care on mechanical ventilation (weaning failure) should be as low as possible. Using a standardized weaning protocol is necessary to achieve this goal. Successful weaning concepts consist of many components: protocols for evaluation and documentation of readiness-to-wean as well as extubation capability, continuous adaptation of sedation goals and adequate analgesia, specialized ventilation modes, and the coordinated approach by an interprofessional team, especially in cases with prolonged weaning. Overall, the weaning QI can be seen in the context of other quality indicators, i.e. ICU rounds with definition of daily goals for weaning (QI I), assessment of analgesia, sedation, and delirium (QI II), patient-adapted ventilation (QI III), measures for infection management, in particular to prevent ventilator-associated pneumonia (QI VI), and early mobilization (QI IX). The formula focusses on stopping invasive ventilation as quickly as possible [23], [24]. Additionally, keeping record of patients discharged to home-care ventilation might be a suitable outcome indicator.

### QI V Monitoring of infection prevention measures

(WG: F. Bloos, A. Brinkmann, P. Czorlich, G. Wöbker)

Multi-resistant pathogens and the persistently high mortality rate from nosocomial infections present a continuing challenge for medical care [25], [26], [27]. Avoiding infections in the ICU is, therefore, an effective measure to reduce morbidity and mortality [28]. The current recommendations of the Commission for Hospital Hygiene and Infection Prevention (KRINKO) for infection prophylaxis and the requirements for hygiene in intensive care published by the Association of the Scientific Medical Societies in Germany (AWMF) were used to develop this indicator [29], [30], [31], [32], [33], p. 205ff. Requirements for structure, process, and outcome are defined. Finally, a formula measures outcome quality. Quality dimensions include appropriate procedural standards and instructions for infection prevention. In addition, as a means of structural quality, participation in the ICU module of the hospital infection surveillance system (KISS) has been added. Sufficient hand hygiene is of special importance and considered fundamental in infection prevention. Therefore, hand disinfectant consumption is measured as approximation for process compliance. However, assessing the effectiveness of individual measures remains difficult. For example, based on a recent Cochrane review from 2020, oral hygiene measures (including use of chlorhexidine) reduce the incidence of VAP, but there is currently no evidence of an effect on mortality, duration of intensive care, and days on mechanical ventilation [34]. An effect on mortality has been observed for selective digestive tract disinfection (SDD) [35], [36] and selective oral disinfection (SOD) [37], but the feasibility and effectiveness of these measures is under discussion [38], [39].

Different measures of outcome quality are recommended. In addition to hand disinfectant consumption, the daily documentation of “stop orders” is now listed as a recommendation. “Stop orders” are part of the KRINKO recommendations for preventing infections caused by foreign materials. As an outcome of the quality indicator, the effectiveness of prevention measures should be monitored based on one or more “marker infections”. These include ventilator-associated pneumonia (VAP), central line-associated bloodstream infection (CLABSI), catheter-associated urinary tract infections (CAUTI), and acute ventriculitis caused by external ventricular drainage (EVD) in patients with neurological or neurosurgical critical illness. The frequency of these “marker infections” should be used to demonstrate the successful implementation of prophylactic measures. The simultaneous monitoring of several marker infections is useful and recommended.

### QI VI Infection management measures

(WG: A. Brinkmann, F. Bloos, G. Wöbker)

This indicator is based on the fact that guideline-based therapy, particularly regarding bacterial infections, still shows potential for improvement. External peer reviews still reveal deficiencies in treatment indication, consideration of organ insufficiencies (liver, kidney) in substance selection, application and dosage of suitable anti-infectious substances, as well as adequate documentation. The indicator, therefore, evaluates two main aspects of treatment: 1. adequate and timely microbiological diagnostics and 2. the use of current guidelines for appropriate anti-infectious therapy [40], [41], [42], [43].

Clinical signs are the basis for diagnosis. In addition to laboratory parameters (i.e. leukocytes, C-reactive protein, procalcitonin, optional interleukin-6) in infections [44], the development of new organ dysfunction is important, as this significantly impairs the patient’s prognosis [41], [43]. This is reflected in the new sepsis criteria [45], in which the assessment of organ dysfunction by the Sequential Organ Failure Assessment Score (SOFA) plays an important role in the ICU. Outside an ICU, monitoring of qSOFA is no longer recommended as stated in the current guidelines of the Surviving Sepsis Campaign (SSC). Different screening tools should be considered there: Systemic Inflammatory Response Syndrome (SIRS), (SOFA) criteria, National Early Warning Score (NEWS) and Modified Early Warning Score (MEWS) [43]. The second essential part of this indicator is appropriate microbiological diagnosis, as reflected in the indicator formula for blood cultures per 1,000 patient days [46], [47]. The evaluation of the therapy process, e.g. in peer review visits, focuses on transparent documentation of indication and duration of anti-infectious therapy. As far as possible, implementation of the described structural and process determinants should be achieved. These include adherence to guidelines, local SOPs [48], [49], [50], [51], [52], timely initiation of therapy [40], [41], [42], [43], multi-professional rounds (microbiologist, clinical pharmacist, infection specialist, etc.) [53], transparent documentation (indication, initiation and duration of therapy), therapeutic drug monitoring (TDM) [54], [55], [56], [57], [58], and antibiotic stewardship (ABS) [59].

In patients with sepsis and septic shock it should be noted that significant changes in the pharmacokinetics of anti-infectious agents may occur, including impairment of drug uptake, distribution, metabolism, and excretion [43], [54], [58], [60], [61]. This results in unpredictable serum drug concentrations in the blood (primary compartment) and consequently also at the site of infection (site of action). This aspect is insufficiently accounted for by traditional dose recommendations. Numerous current guidelines, therefore, recommend individual dosing concepts, especially for patients with sepsis and septic shock [40], [41], [43], [54], [60]. These can be supported by TDM, but also with digital tools (see tabular view of QI in Attachment 1). In addition to improving the efficacy of anti-infectious agents, it is also important to reduce adverse drug effects (e.g. neurotoxicity and nephrotoxicity) [43], [54], [58], [60], [61]. Prolonged [62] or TDM-controlled continuous [60] administration of beta-lactam antibiotics to enhance efficacy and continuous administration of vancomycin to reduce nephrotoxicity are supported by current guidelines [40], [41], [43] and the literature [63], [64], [65].

### QI VII Patient-adapted clinical nutrition

(WG: O. Kumpf, E. Muhl, A. Schäfer)

Almost all intensive care patients require early clinical nutritional therapy since malnutrition, severe obesity, severe metabolic disorder, or substrate utilization disorder are often present. The goal is to start an individualized clinical nutritional therapy – preferably administered enterally – at an early stage. Screening for malnutrition, setting individual nutritional goals, and monitoring of the effectiveness of therapy are key for success. Clinical nutrition therapy is based on the current guidelines of the German Society for Nutritional Medicine (DGEM) [66]. A multi-professional standard based on this current S2k-guideline should be defined at each ICU. For individualized nutritional therapy, the determination of an adapted caloric target (e.g. in patients with severe obesity or malnutrition) should be present. This caloric target is based on a patient’s body weight, nutritional status, and the treatment situation.

### QI VIII Structured communication with patients and relatives

(WG: M. Brauchle, J.-P. Braun, A. Brinkmann, P. Czorlich, O. Kumpf, M. Ufelmann, R. Wildenauer)

Treatment in the ICU, whether elective or by emergency admission, must be consistent with the patient’s will. The patient’s expectations and goals must be congruent with the goals of intensive care treatment. In its course it is therefore necessary to re-evaluate planned and achieved treatment results by considering the patient’s will, in order to avoid potential harm to the patient, but also to relatives or the members of the ICU-team. To achieve this balance, structured discussions with patients, their next of kin, or legal guardians are very important. The success of those discussions depends on their structure and the qualification of physicians and nurses [67]. The communication itself and the documentation should follow recommendations for structure [68]. The use of special forms or templates in the PDMS is recommended. Within 72 hours of admission to the ICU, an initial conversation should take place. Follow-up communication should happen at least once a week. Ideally, all disciplines and professions involved in the treatment should participate. In this discussion the current status of the patient has to be considered as well as the patient’s will, the prognosis, the probability of a treatment success, and treatment options and its consequences, followed by the proposal of a treatment plan [69]. The structure of this discussion should follow current recommendations (e.g. the VALUE concept, see QI table in Attachment 1). Results of the communication should be explained to the ICU-team and documented. In addition, the use of patient diaries to support family members is recommended. Surveys among patients’ family members as feedback can help to identify and subsequently address communication deficiencies in an ICU [70].

### QI IX Early mobilization

(WG: R. Dubb, A. Kaltwasser, P. Nydahl, S. J. Schaller)

There is an increasing number of critically ill patients who need long-term ventilator support, so any measure to avoid long-term ventilator dependence is useful (see also QIs II and IV). The positive effects of early mobilization in terms of shorter periods of mechanical ventilation and shorter intensive care stay are well documented [71], [72], [73]. There is also evidence of a lower incidence of delirium, improved muscle strength and independence in critically ill patients, and more days alive outside the hospital within 180 days [71], [74], [75], [76]. The indicator emphasizes an early start of mobilization, based on defined standards in an ICU [71]. Mobilization inside and outside of the bed is carried out according to transparent criteria for inclusion, exclusion, and safety [76], [77], [78]. In addition, it is important to avoid immobilization, which should be ordered explicitly.

### QI X Direction of the intensive care unit

(WG: J. Braun, A. Brinkmann, P. Czorlich, R. Dubb, A. Kaltwasser, O. Kumpf, A. Markewitz, G. Marx, E. Muhl, S. Pelz, R. Riessen, R. Wildenauer, G. Wöbker, H. Wrigge)

At the time of publication, this indicator will be revised according to the recently published recommendations of the DIVI for the structure of intensive care units. Therefore, this indicator remains unchanged at the moment. For the adequate care of critically ill patients, the 24-hour presence of an experienced and qualified nursing and medical team is necessary. The term “presence” may include short-term assignments outside the ICU, e.g. for emergency care of patients within the hospital (resuscitation service, MET), but not obligations in other clinical or non-clinical care areas. Studies show that during core working hours, i.e. the time when important decisions are made in an interdisciplinary and interprofessional context and when interdisciplinary specialists are available, the presence of an experienced and qualified intensive care physician is necessary [79], [80]. This intensive care physician may not combine any major (clinical) tasks other than the professional management of the ICU. This corresponds to the relevant structural requirements defined by DIVI [81]. The indicator is also evaluated based on these structural specifications for intensive care. Adequate staffing can only be ensured by close contact between the medical, the nursing and the organizational management of a hospital. Staffing is based on the structural needs of individual ICUs and hospitals and may depend on externally provided services (i.e. dialysis, transport, material supply, etc.).

## Notes

### Acknowledgement

The authors wish to thank Volker Parvu for his support in the preparation of the quality indicators.

### Competing interests

The authors declare that they have no conflicts of interest in connection with this article. Potential conflicts of interest for QI development are indicated in Attachment 2.

## Supplementary Material

Tabular view of the QIs

Potential conflicts of interest
