# Supplementary material for: Quality indicators in intensive care medicine for Germany – fourth edition 2022
Source: Ger Med Sci. 2023 Jun 23;21:Doc10. doi: 10.3205/000324 (PMC10326525; doi:10.3205/000324)
Supplement: Tabular view of the QIs [file GMS-21-10-s-001.pdf]

Attachment 1

Comparison of the quality indicators of DIVI 2017 and 2022

a) Quality indicators intensive care (4th edition 2022)

Number	Main Indicators I–X
I	Daily multi-professional and interdisciplinary ICU rounds with documentation of daily goals
II	Management of sedation, analgesia, and delirium
III	Patient-adapted ventilation (for severe lung failure)
IV	Early weaning from invasive ventilation
V	Monitoring of infection prevention measures
VI	Infection management measures
VII	Patient-adapted clinical nutrition
VIII	Structured communication with patients and relatives (next of kin)
IX	Early mobilization
X	Direction of the intensive care unit

b) Quality indicators intensive care (3rd edition 2017)

Number	Main Indicators I–X
I	Daily multi-professional and interdisciplinary clinical visits with documentation of daily goals
II	Management of sedation, analgesia and delirium
III	Patient-adapted ventilation
IV	Early weaning from invasive ventilation
V	Monitoring of infection prevention measures
VI	Measures for infection management
VII	Early enteral nutrition
VIII	Documentation of structured patient and family communication
IX	Early mobilization
X	Direction of the intensive care unit

Attachment 1 to Kumpf O, Assenheimer M, Bloos F, Brauchle M, Braun JP, Brinkmann A, Czorlich P, Dame C, Dubb R, Gahn G, Greim CA, Gruber B, Habermehl H, Herting E, Kaltwasser A, Krotsetis S, Kruger B, Markewitz A, Marx G, Muhl E, Nydahl P, Pelz S, Sasse M, Schaller SJ, Schäfer A, Schürholz T, Ufelmann M, Waydhas C, Weimann J, Wildenauer R, Wöbker G, Wrigge H, Riessen R. Quality indicators in intensive care medicine for Germany – fourth edition 2022. GMS Ger Med Sci. 2023;21:Doc10. DOI: 10.3205/000324, URN: urn:nbn:de:0183-0003244

The new quality indicators of the DIVI in tabular form

Main indicator I

Name of the indicator	**Daily multi-professional and interdisciplinary ICU rounds with documentation of daily goals**
Dimension	Risk and effectiveness
Type of indicator	Process
Justification	Daily multi-professional and interdisciplinary ICU rounds improve communication among the different professions involved in ICU treatment. Of particular importance is the written documentation of daily goals for each patient. The determination of daily (short-term) and longer-term objectives should lead to more effective implementation of planned measures considering treatment objectives and thus improve the quality of treatment. There is a risk of information loss and thus loss of quality due to poorly structured handovers, visits, and their documentation.
PICO	Performing a daily multi-professional and interdisciplinary ICU round with documentation of daily goals improves communication among the ICU treatment team and improves the outcome of this treatment compared to an unstructured ICU round.
Quality goal	Permeating the daily routine in an ICU with predefined daily goals and carrying out a multi-disciplinary visit
Process quality numerator	Daily visits with comprehensibly documented treatment goals
Denominator	All treatment days of a patient in the ICU
Explanation of terminology	**ICU round:** Daily interprofessional and – depending on the treatment spectrum of the ICU – also interdisciplinary ICU round with at least one senior ICU-physician responsible for all medical decisions (head of the ICU or other senior ICU-physician) present. The ICU rounds should enable all participating professions to provide and receive information regarding the patient's clinical status.<br>**Daily goals:** Daily goals should be defined during the ICU round, involving all professions and disciplines, including the adequate indication of a treatment.<br>The following points should be considered when defining daily goals:<br>• Coordination of communication (consultants/relatives/institutions providing treatment) (see also QI VIII)<br>• Re-evaluation of therapy goals/change of therapy goals/ethical decisions<br>• Analgesia, sedation and delirium prevention and management<br>• Mechanical ventilation/Weaning/SAT/SBT (see QI III/IV)<br>• Hemodynamic situation/fluid balance<br>• Nutrition (see QI VII)<br>• Infection management (see QI V/VI)<br>• Definition of preventive measures (anti-coagulation/decubitus/gastric protection/ early mobilization/special physiotherapy measures)<br>• Planned measures (diagnostic/therapeutic); do they have a consequence in the treatment of the patient?<br>• Adaption of medication<br>**Documentation:** The more professions or disciplines are involved in the treatment of a patient, the more difficult it is to unite them on one ICU round. Therefore, written statements are very important to ensure information flow. A written documentation shows what was defined by the primary participants and helps others who were unable to attend to comprehend what was considered important. Modifications of therapeutic goals can be understood and followed.<br>Documentation templates support the communication-enhancing effect of a multi-professional and interdisciplinary ICU round.<br>Daily goal checklists support the implementation as has been shown in the literature. However, checklists alone do not improve patient safety.

Source of data	Patient record, PDMS<br>Query: peer review
Standard value	**90% correctly documented ICU rounds**
Level of evidence	Expert consensus
QUALIFY	Optional evaluation
WG-members	R. Wildenauer, A. Brinkmann, A. Markewitz, M. Assenheimer
Conflicts of interest	See Attachment 2
Literature	[1, 2–11]
Additional information	Validated daily targets/items (acronyms):<br>• A: Pain management<br>• B: SBT – SAT<br>• C: Analgesia – sedation<br>• D: Delirium prevention and management<br>• E: Early mobilization – physiotherapy<br>• F: Involvement of the family<br>TRIKK: 5 guiding questions, which can be easily answered during the visits – guide to the patient's wishes (acronym):<br>• Formulate the therapy goal (T)<br>• Re-evaluate the therapy goal regularly (R)<br>• Is there an appropriate indication? (I)<br>• The planned procedure has a consequence and serves the therapeutic objective (K)<br>• Is there a consensus of the patient for the current or planned treatment? (K)

Main indicator II

Name of the indicator	**Management of sedation, analgesia, and delirium**
Dimension	Risk and effectiveness
Type of indicator	Process
Justification	Inadequate sedation (oversedation or undersedation), inadequate analgesia, and untreated delirium result in increased morbidity, mortality, and resource consumption. A multi-modal concept for the management of analgesia, sedation and delirium should be available as a standard in every ICU. Regular monitoring of sedation depth and analgesia quality as well as the use of instruments to detect delirium are prerequisites for the implementation of such a concept.<br>Indicator II is divided into the **management of sedation, analgesia, and delirium**.<br>**Structure:** Are there SOPs that cover all three aspects (sedation, analgesia, and delirium)?<br>**Process:** Once per shift, a validated score is collected for analgesia, sedation, and delirium. Measuring these scores represents only a small technical and time-consuming effort; therefore, only one missing value is acceptable. The reference value has been adjusted accordingly.<br>Optional evaluation of **outcome quality:** An analysis is **recommended** at least once a year; institutions using a PDMS may use shorter intervals.<br>a) Sedation (periods without sedation, times in the target range +/–1)<br>b) Analgesia (percentage of pain score in target range)<br>c) Delirium (only prevalence assessment; was a therapy initiated, if yes, what therapy?)
PICO	Can a regular, continuous measurement of sedation, pain and delirium scores improve the treatment process in these dimensions in adult intensive care patients compared to no score measurement?

Quality goal	Ensure timely and continuous monitoring of inappropriate sedation, inadequate pain management and detection of delirium throughout the course of ICU treatment
Structure quality	A standard for the management of sedation, analgesia and delirium is available
Process quality numerator	Number of all score-measurements performed
Denominator	Total number of possible measurements in all ICU patients during the entire treatment period<br>(Total number of predefined measurements = (treatment days –1) x3)
Explanation of terminology	The use of validated sedation, analgesia and delirium scales is recommended in clinical guidelines.<br>Monitoring: assessment of sedation and analgesia levels as well as presence of delirium on validated scales every 8 hours or if the clinical situation changes.<br>Recommended scales [SCORE]:<br>• RASS: Richmond Agitation and Sedation Scale<br>• NRS: Numeric Rating Scale or BPS: Behavioral Pain Scale<br>• CAM-ICU: Confusion Assessment Method – Intensive Care Unit<br>• ICDSC: Intensive Care Delirium Screening Checklist<br>or other validated delirium scores.<br>Other aspects of a concept for managing analgesia, sedation and delirium include the use of preventive measures (e.g. maintaining the day-night rhythm) and non-pharmacological therapies. The basic avoidance of sedation and the daily interruption of a long-term sedation (wake-up call) should be included in a standard. The definition of sedation targets and, if necessary, the technical monitoring of the sedation depth when deep sedation is indicated are additional aspects of management. Providing information materials for relatives may help to implement these concepts.
Source of data	1. Structure: query<br>2. Process: patient record (clinical documentation); PDMS<br>3. Outcome: patient record (clinical documentation); PDMS<br>Query: peer review
Standard value	1. Structure (SOPs: sedation/analgesia/delirium)<br>Standard yes/no (Yes=100%)<br>2. Process scoring (sedation/analgesia/delirium):<br>Scoring frequency ≥88% (missing one measurement)<br>3. Outcome (optional): (no specifications)<br>Target-performance comparison (sedation/analgesia/delirium)
Level of evidence	Expert consensus
QUALIFY	Optional evaluation
WG-members	O. Kumpf, P. Czorlich, S. Krotsetis
Conflicts of interest	See Attachment 2
Literature	[12]

Additional information	Scoring algorithm<br>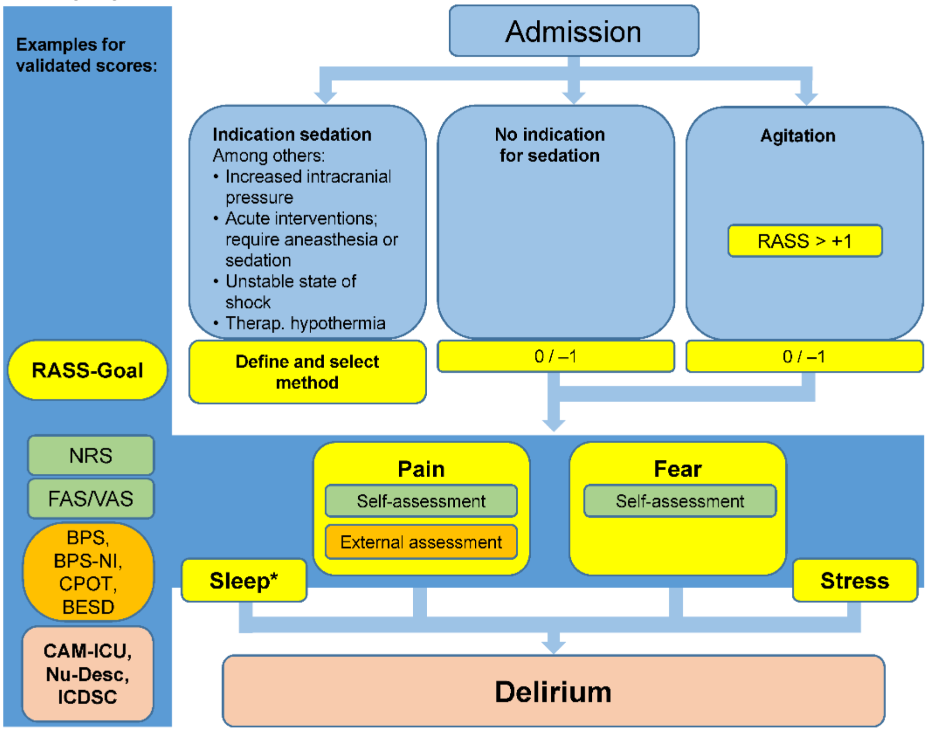<br><br>**Implementation help for DAS management with examples of validated measurement scales:**<br>**RASS:** Richmond Agitation Sedation Scale, **NRS:** Numerical Rating Scale, **VAS:** Visual Analogue Scale, **BPS:** Behavioral Pain Scale, **BESD:** Assessment of Pain in Dementia, **FAS:** Faces Anxiety Scale, **CAM-ICU:** Confusion Assessment Method for the Intensive Care Unit, **ICDSC:** Intensive Care Delirium Screening Checklist, **Nu-Desc:** Nursing Delirium Screening Scale;<br>*Polysomnography: validated, but too complex, sleep monitoring apps available, not validated in ICU setting<br>Algorithm translated from: Wolf A., Müller A, Weiß B, Spies C. S3-Leitlinie: Delir-, Analgesie- und Sedierungsmanagement in der Intensivmedizin. Anästh Intensivmed 2016;57:41–44.

Main indicator III

Name of the indicator	**Patient-adapted ventilation (for severe lung failure)**
Dimension	Risk and effectiveness
Type of indicator	Process
Justification	Severe acute pulmonary failure (ARDS) requires ventilation and positioning strategies adapted to the individual patient in order to prevent further pulmonary and systemic damage and to improve outcome. ARDS is often diagnosed too late or not at all, which leads to insufficient treatment because standards are not consistently applied. A standardized, stepwise approach to ventilator therapy (i.e. a standard for mechanical ventilation) for severe acute pulmonary failure can improve survival and should be present in every ICU.<br>The concept should describe a stepwise approach based on the current S3-guideline for invasive ventilation and extracorporeal lung support of the AWMF registry. It should include different aspects of optimizing mechanical ventilation (e.g. PEEP optimization, limitation of pulmonary strain and driving pressure ($delta_P$=V_T/Compliance)) and adjunctive therapies such as prone positioning. Early contact to a specialized center for ARDS therapy should be possible, unless extracorporeal lung support is performed on a particular ICU.
PICO	Does the use of standardized mechanical ventilation reduce morbidity and mortality in adult patients with severe acute pulmonary failure (ARDS) compared to treatment without a standard?
Quality goal	The indicator is intended to improve the treatment outcome of severe respiratory failure by applying standardized treatment procedures based on the most recent scientific evidence in ARDS therapy. The focus is an individualized mechanical ventilation strategy.

Structure quality	Mechanical ventilation standard is available: yes/no
Process quality numerator	Number of patients with severe pulmonary failure and treatment according to a guideline-based mechanical ventilation standard
Denominator	All mechanically ventilated patients with pulmonary failure
Explanation of terminology	The aim of patient-adapted ventilation is to ensure an adequate (not necessarily physiological) gas exchange while avoiding secondary pulmonary parenchymal damage and systemic inflammation (biotrauma). Adjunctive measures such as prone positioning may support this in the treatment of severe ARDS.<br>Components of the standard for a patient-adapted therapy should include limiting tidal volume (V_T 6–7 ml/kg ideal body weight), limiting plateau pressure (30 cmH_2O), and individualized PEEP setting. Driving pressure ($Delta_P$) considers compliance in addition to V_T and is thus more suited for estimating the mechanical stress of ventilation than V_T alone. $Delta_P$>14–15 cmH_2O are associated with excess mortality.<br>Individualization of PEEP can be pragmatically based on the extent of hypoxemia (e.g. PEEP table). Furthermore, patient-related factors (e.g. preexisting pulmonary damage, obesity, and the hemodynamic state) should be considered. This may include bedside measurements (e.g. driving pressure, transpulmonary pressure, HZV) and diagnostic imaging (e.g. chest computed tomography, ultrasound, electric impendance tomography=EIT). Recruitment maneuvers should not be performed. Prone positioning is recommended for patients with a PaO_2/F_IO_2<150mmHg. In the absence of contraindications (e.g. increased intracranial pressure), spontaneous ventilation should be allowed early, as this leads to improved basal lung ventilation, needs less sedation, and improves hemodynamics. Use of muscle relaxants is not recommended (any more).<br>If necessary, a transfer process to a specialized center for the treatment of severe ARDS with extracorporeal lung support should be established.
Source of data	1. Structure: standard/SOP available<br>2. PDMS, patient file<br>Query: peer review
Standard value	1. Structure<br>yes 100% (SOP – ventilation standard)<br>2. Process:<br>≥70% patient-adapted ventilation
Level of evidence	Expert consensus
QUALIFY	Optional evaluation
AG-members	T. Schürholz, H. Wrigge, B. Kruger, O. Kumpf
Conflicts of interest	See Attachment 2
Literature	[13, 14, 15–21]

Main indicator IV

Name of the indicator	**Early weaning from invasive ventilation**
Dimension	Risk and effectiveness
Type of indicator	Process
Justification	Invasive ventilation is associated with the risk of complications (e.g. ventilator-associated pneumonia (VAP), ventilation-induced lung injury (VILI), delirium, and atrophy of the respiratory muscles). The therapeutic goal is to achieve the shortest possible duration of mechanical ventilation by early weaning of invasive ventilation, depending on the status of the underlying disease.<br>Depending on type and severity of the disease, application of non-invasive ventilation (NIV) or the application of oxygen via high flow nasal canula (HFNC) should be considered. This helps to avoid invasive ventilation or reintubation after primary successful extubation.
PICO	Structured weaning with a protocol shortens the length of mechanical ventilation and reduces the number of patients with unsuccessful prolonged weaning compared to weaning without a protocol.
Quality goal	Lowest possible number of patients with unsuccessful weaning
Structure quality	Existence of a guideline-based concept (SOP) for weaning, in particular for prolonged weaning and a structured weaning documentation in the patient file
Process quality numerator	Number of patients with invasive mechanical ventilation with weaning evaluation and/or a documented weaning process
Denominator	All patients with invasive mechanical ventilation
Outcome quality	Number of mechanically ventilated patients transferred to out-of-hospital treatment after unsuccessful weaning (optional survey)
Explanation of terminology	**Structure and process characteristics:** Daily check of prerequisites for weaning and a spontaneous breathing trial, using a standardized weaning protocol. There is also a connection to QI II, which addresses daily sedation targets with documentation of score values. A guideline-based concept (e.g. SOP) for weaning, especially for prolonged weaning, should be implemented by an interprofessional treatment team (physicians, nurses, physiotherapist, respiratory therapist, speech therapist). A comprehensible weaning documentation in the medical file, incl. documentation of a weekly weaning-conference is necessary. This facilitates the implementation of the newly created weaning OPS for reimbursement purposes.
Source of data	Structure: standard/SOP weaning concepts available<br>Process: PDMS, clinical documentation<br>Outcome: PDMS, controlling data<br>Query: peer review
Standard value	Structure: yes/no – yes 100%<br>Process: >70% number of positive responses
Level of evidence	Expert consensus
QUALIFY	Optional evaluation
AG-members	R. Riessen, H. Habermehl, O. Kumpf
Conflicts of interest	See Attachment 2
Literature	[15, 22, 23]

Main indicator V

Name of the indicator	**Monitoring of infection prevention measures**
Dimension	Risk and effectiveness
Type of indicator	Structure, process, outcome
Justification	Patients in the ICU are at high risk of hospital acquired infections. This becomes more and more important with the increasing occurrence of multi-resistant pathogens (MRE). Within the framework of the Infection Protection Act, medical institutions are highly responsible for prevention of those infections.<br>**1. Structural quality:**<br>For the implementation of effective infection prevention, established hygiene standards (e.g. KRINKO recommendations, S1 guidelines of the AWMF) must be followed. These standards include hand disinfection, treatment of patients with multi-resistant pathogens, VAP prophylaxis, hygiene measures for invasive procedures, etc.). These measures should be documented in an SOP for infection prevention in an ICU and should reflect these current recommendations. It is recommended that infection prevention strategies are bundled and developed by multi-disciplinary and interprofessional teams adapted to local conditions.<br>**2. Process quality:**<br>• Adequate hand hygiene is a fundamental part of the prevention of nosocomial infections. Therefore, the German campaign “Aktion Saubere Hände” was launched based on the WHO campaign “Clean Care is Safer Care” to improve compliance with hand disinfection. This compliance can be monitored indirectly by measuring hand disinfectant consumption.<br>• The indication of catheters (e.g. leaving in place, new implantation) should be documented daily in the patient records (“stop orders”).<br>**3. Outcome quality**<br>• Ventilator-associated pneumonia (VAP)<br>• Catheter-associated urinary tract infections (CAUTI)<br>• Central Line-Associated Bloodstream Infection (CLABSI)<br>• External ventricular drainage (EVD) associated ventriculitis<br>are typical infections complicating ICU treatment. Recommendations for their prevention should be followed appropriately. Monitoring the frequency of at least one of these infections (surveillance) provides the opportunity to identify problems in hygiene management and to measure the success of a quality improvement measure. Participation in the ITS-KISS module of the National Reference Center for Surveillance of Nosocomial Infections (NRZ) is a suitable tool to support the documentation of the quality of results.
PICO	Can the use of standard operation procedures for prevention of nosocomial infection and infection surveillance reduce the incidence of nosocomial infections compared to the absence of these measures?
Quality goal	The quality indicator monitors the structure, process, and outcome quality as a measure for the implementation of infection prevention guidelines. The aim is to ensure that current recommendations for infection prevention are implemented in ICUs.
Structure quality	Implemented standard operation procedure for infection prevention
Process quality	
Numerator	Number of STOP ORDERS
Denominator	Number of invasive devices
Numerator	Hand disinfectant consumption in liters
Denominator	1,000 patient days
Outcome quality	ITS-KISS of the NRZ
Numerator	Number of infection events per device
Denominator	1,000 device days

Explanation of terminology	**5 indications of hand disinfection:**<br>1. BEFORE patient contact<br>2. BEFORE an aseptic activity<br>3. AFTER contact with potentially infectious materials<br>4. AFTER patient contact<br>5. AFTER contact with the immediate patient environment<br><br>**Possible measures for VAP prophylaxis**<br>For this purpose, various measures are mentioned in the literature that can contribute either as a bundle of measures (VAP bundle) or as a single measure to reduce the incidence of VAP. The composition of the VAP bundle differs in the literature. Therefore, the impact of individual bundle components on outcome is difficult to assess. However, VAP bundles as such have been shown to reduce the incidence of VAP. It is recommended to have implemented at least three measures of a VAP bundle in the standards of the ICU, e.g.: oral care and oral decontamination with antiseptic solutions (ODD), avoidance of oral aspiration, e.g. by regular cuff pressure measurements, and continuous subglottic secretion suction. There is evidence that oral hygiene containing chlorhexidine reduces the VAP frequency but does not affect the further course (e.g. duration of respiration, ICU length of stay, mortality). There is a lack of data regarding possible adverse effects.<br><br>**Possible measures for CLABSI prophylaxis**<br>It is recommended to have SOPs for the implantation and care of intravascular catheters and to train their use. Measures for catheter placement should include: hand disinfection before puncture, choice of skin disinfection (e.g. chlorhexidine-containing solutions), maximum sterile barrier precaution (sterile gloves, sterile coat, mask, sufficiently large sterile cover), and information on puncture techniques (avoidance of V. femoralis as a puncture site, sonography). Catheter care should include information on disinfection when using the catheter, indications for use (avoidance of unnecessary manipulation), catheter removal, and care of the insertion site.<br><br>**Possible measures for CAUTI prophylaxis**<br>It is recommended to have SOPs for the installation and care of urinary bladder catheters and to train their use. Catheter insertion measures should include: indication and daily inspection, aseptic placement of the catheter, use of sterile and closed urinary drainage systems, management of the catheter in place, instructions for emptying the bag, and management of catheter removal.<br><br>**Possible measures for the prophylaxis of external ventricular drainage (EVD) associated ventriculitis**<br>It is recommended to have SOPs for the installation and maintenance of drainage systems and to train their use. Installation of drainage systems in the operating room as far as possible. Measures for the installation of the drains should include: hand disinfection before puncture, choice of skin disinfection (e.g. chlorhexidine-containing solutions), maximum sterile barrier precaution (see above), and microbiological monitoring of the cerebrospinal fluid every 2–3 days and depending on clinical signs. Daily monitoring (and documentation) of the insertion site.
Source of data	1. Structure: query<br>2. Process: patient record; PDMS<br>3. Patient file or KISS data<br>Query: peer review
Standard value	Structure:<br>• SOPs for infection prevention available<br>• Participation in the ITS-KISS module of the NRZ<br>Process:<br>• Hand disinfectant consumption >80–100 liters/1,000 patient days<br>• Daily stop orders established in the patient record, indication documented. Stop orders >80%<br>Result:<br>• Decreasing rate of nosocomial infections over time based on selected lead infection

Level of evidence	Expert consensus
QUALIFY	Optional evaluation
AG-members	F. Bloos, A. Brinkmann, G. Wöbker, P. Czorlich
Conflicts of interest	See Attachment 2
Literature	[24–31]

Main indicator VI

Name of the indicator	**Infection management measures**
Dimension	Risk and effectiveness
Type of indicator	Structure, process and outcome
Justification	Early, adequate, and effective diagnosis of infections, anti-infectious therapy and the effective prevention of resistance development and collateral damage, especially in the gastrointestinal microbiome, are of paramount importance for the management of infections in the ICU.<br>The following principles should be followed:<br>• Early and adequate empiric antibiotic therapy in patients with severe infections and organ failure (sepsis and septic shock); precise microbiological diagnosis and targeted therapy is carried out in patients with low disease severity<br>• Adequate microbiological diagnosis with suitable materials (including blood cultures) before starting antibiotic therapy<br>• Targeted measures to avoid unnecessary anti-infectious treatments as well as unnecessary and prolonged antibiotic prophylaxis<br>For the implementation of reliable and effective infection management, an interdisciplinary and interprofessional collaboration including a well-established antibiotic stewardship (ABS) team is recommended.
PICO	Can the application of standard operating procedures and the monitoring of the appropriate application of guidelines and monitoring of pathogen diagnostic methods improve the outcome of treatment for patients with sepsis compared to non-standardized and monitored diagnostics and therapy?
Quality goal	Early, adequate, and effective diagnosis of infection, source control, and anti-infectious therapy significantly contribute to improved outcome (mortality, complications and duration of treatment) in critically ill patients with severe infections, sepsis, and septic shock. Rational, targeted, and reliable use of anti-infectious substances also significantly reduce the development of microbial resistance and treatment costs
Structure quality	SOP for infection management available
Process quality numerator	Number of adequate antibiotic therapies
Denominator	All ICU supervised and treated patients with DRG code (for sepsis)
Process quality numerator	Number of adequate documentation (indication, focus, expected duration of therapy, current guidelines)
Denominator	All ICU supervised and treated patients with DRG code
Outcome quality numerator	Number of blood cultures taken
Denominator	1,000 patient days
Explanation of terminology	The new definition of sepsis in 2016 moves the focus on clinical aspects. In addition, the SOFA score plays an important role in the detection of sepsis. It is recommended to record the SOFA score daily in all patients with relevant sepsis risk.<br>Methods to improve anti-infectious therapy in the ICU setting include:<br>Structure quality:<br>• ABS team (ABS expert, hygiene specialists, infectious disease specialist, ICU physician, clinical pharmacist, microbiologist)<br>• Local guidelines for antibiotic use and SOPs<br>• Local microbial-resistance statistics

	• Therapeutic drug monitoring<br>• Surveillance of anti-infective drug consumption<br>Process quality:<br>• Adherence to current national guidelines for antimicrobial therapy as well as diagnosis and therapy of sepsis. S2k-LL PEG (2018 update), S3-LL DGI (2018 update), S3-LL sepsis (2020 update), Surviving Sepsis Campaign (SSC 2021 update)<br>• Early and adequate microbiological diagnosis (including blood cultures 2-3 x 2) before starting therapy (SOP)<br>• Timely focus control<br>• Prompt (<1 h) and adequate anti-infective therapy (SOP)<br>• De-escalation at the earliest possible stage<br>• Multi-professional rounds (intensive care specialist, hygienspecialists, infectious disease specialist (if available), ICU-physician, clinical pharmacist, microbiologist) see also QI I<br>• Transparent documentation of indication and expected duration of therapy<br>• Use of therapeutic drug monitoring (TDM, mandatory for aminoglycosides and glycopeptides, recommended for beta-lactams, linezolid, and voriconazole)<br>• Consideration of pharmacokinetic and pharmaco-dynamic principles in dosing and administration, e.g. prolonged/continuous (only with near-time TDM [results available <24 h]) of beta-lactam antibiotics and vancomycin<br>• Use of dose calculation tools for antibiotic dosing in the presence of impaired renal function (see additional information)<br>• Considering drug incompatibilities<br>• Implementation of ABS in daily clinical practice<br>Outcome quality:<br>Monitoring of appropriate indicators to evaluate the effectiveness of the given standards:<br>• Number of blood cultures/1,000 patient days<br>• Proportion of adequate (following current GL) anti-infective therapies<br>• Proportion of adequate documentation (indication, focus and expected duration of therapy)
Source of data	1. Hospital information system, PDMS<br>2. ICU KISS (NRZ)<br>3. Routine DRG data<br>Query: peer review
Standard value	1. Number of blood cultures ≥80/1,000 patient days<br>2. Number of adequate antibiotic therapy >80%<br>3. Number of adequate documentation (indication, focus, expected duration of therapy, current guidelines) >90%
Level of evidence	Expert consensus
QUALIFY	Optional evaluation
AG-members	A. Brinkmann, F. Bloos, G. Wöbker
Conflicts of interest	See Attachment 2
Literature	[29–43]
Additional information	Use of dose calculation tools for dose finding in impaired renal function.<br>Calculation tools:<br>• www.dosing.de<br>• www.clincalc.com<br>• www.tdmx.eu<br>• www.thecaddy.de<br>• VancoEasy/MeroEasy (downloadable apps)

Attachment 1 to Kumpf O, Assenheimer M, Bloos F, Brauchle M, Braun JP, Brinkmann A, Czorlich P, Dame C, Dubb R, Gahn G, Greim CA, Gruber B, Habermehl H, Herting E, Kaltwasser A, Krotsetis S, Kruger B, Markewitz A, Marx G, Muhl E, Nydahl P, Pelz S, Sasse M, Schaller SJ, Schäfer A, Schürholz T, Ufelmann M, Waydhas C, Weimann J, Wildenauer R, Wöbker G, Wrigge H, Riessen R. Quality indicators in intensive care medicine for Germany – fourth edition 2022. GMS Ger Med Sci. 2023;21:Doc10. DOI: 10.3205/000324, URN: urn:nbn:de:0183-0003244

Main indicator VII

Name of the indicator	**Patient-adapted clinical nutrition**
Dimension	Risk and effectiveness
Type of indicator	Structure and process
Justification	Almost all patients in the ICU require timely clinical nutritional therapy. This may be due to malnutrition, severe obesity, a severe metabolic disorder, or abnormal substrate utilization. Early initiation of individualized clinical nutrition therapy based on a defined nutritional goal is associated with lower mortality in intensive care patients. The preferred route of application is enteral. Parenteral nutrition may be used as a supplement. Clinical nutrition therapy is based on the current guidelines of the German Society for Nutritional Medicine (DGEM). Nutritional therapy starts with screening for malnutrition, then sets individual nutritional goals and monitors the effectiveness of nutritional therapy. Current metabolic status, stage of disease, and substrate requirements have to be considered. Every ICU should have a standard for nutritional therapy. It describes the aspects mentioned above.
PICO	Can individualized clinical nutrition therapy based on an established standard positively influence the course of critical illness and patient-centered outcomes compared to a purely quantitatively based therapy?
Quality goal	ICU patients receive a standard-based nutritional therapy adapted to their individual needs. The use of the quality indicator is intended to minimize the number of patients receiving inadequate nutritional therapy.
Structure quality	Nutritional standard available
Process quality numerator	Number of patients receiving enteral nutrition within 24 h
Denominator	Number of all patients in whom adequate oral nutrition was not foreseeable from day 1
Process quality numerator	Number of patients with BMI ≤30 who received adequate nutrition based on the calculated calorie target and adjusted to the patient's metabolism and who reached this dietary target
Denominator	Number of all patients receiving nutrition with BMI ≤30
Explanation of terminology	• Standard: A multi-professional standard should be defined by consensus based on the current DGEM-guideline.<br>• Individualised: Determination of the caloric target, e.g. by calculating it using simple formulas (24 Kcal/kgBW/day, in patients up to a BMI of 30). Individual control of dietary therapy should be based on metabolic requirements (especially insulin dose and serum phosphate concentration) (see figures below).<br>• Patients with severe obesity or malnutrition should be addressed in the SOP.<br>• Early: Start of clinical nutrition therapy within 24 hours of admission if adequate oral feeding is not foreseeable.<br>• Interruptions (e.g. after reflux measurements or before interventions etc.) should be avoided.<br>• Enteral vs. parenteral: All patients without contraindication should receive primarily enteral nutrition. If enteral nutrition is limited or not possible, supplemental or complete parenteral nutrition should be provided.<br>• Calorie target (stage-dependent): The calorie target is based on the patient's body weight and nutritional status. The current guidelines of the professional societies do not show a consensus on the appropriate amount of calories. If possible, start with 75% of the identified daily requirement and reach the nutritional target of 100% after the acute phase.
Source of data	1. Query<br>2. Process: patient file/PDMS<br>3. Process: patient record/PDMS<br>Query: peer review
Standard value	1. Structure: yes/no (SOP available) (yes=100%)<br>2. Process: early start in >90% of patients<br>3. Process: number of adequately fed patients >70%

Attachment 1 to Kumpf O, Assenheimer M, Bloos F, Brauchle M, Braun JP, Brinkmann A, Czorlich P, Dame C, Dubb R, Gahn G, Greim CA, Gruber B, Habermehl H, Herting E, Kaltwasser A, Krotsetis S, Kruger B, Markewitz A, Marx G, Muhl E, Nydahl P, Pelz S, Sasse M, Schaller SJ, Schäfer A, Schürholz T, Ufelmann M, Waydhas C, Weimann J, Wildenauer R, Wöbker G, Wrigge H, Riessen R. Quality indicators in intensive care medicine for Germany – fourth edition 2022. GMS Ger Med Sci. 2023;21:Doc10. DOI: 10.3205/000324, URN: urn:nbn:de:0183-0003244

<table>
<tr><td>Level of evidence</td><td>Expert consensus</td></tr>
<tr><td>QUALIFY</td><td>Optional evaluation</td></tr>
<tr><td>AG-members</td><td>O. Kumpf, E. Muhl, A. Schäfer</td></tr>
<tr><td>Conflicts of interest</td><td>See Attachment 2</td></tr>
<tr><td>Literature</td><td>[44, 45]</td></tr>
<tr><td>Additional information</td><td>
<table>
<tr><th></th><th>Day 0</th><th>Day 1</th><th>Day 2</th><th>Day 3 onward</th></tr>
<tr><td>Nutrition substrate</td><td>None</td><td>75% of predetermined caloric goal incl. 0,75 g/kg/BW protein (=3 kcal/kg/BW)</td><td>24 kcal/kg/BW if phos-phate level >0.65 mmol/l and insulin = <1 IE/h</td><td>…</td></tr>
<tr><td>Insuline requirements</td><td>--</td><td>--</td><td>2–4 IE/h
→ 12/kcal/kgBW/day
>4IE/h
→ 6/kcal/kgBW/day</td><td>If persistent, reduce substrate further (minimum 0 kcal/d)</td></tr>
<tr><td>Phosphate concentration</td><td>--</td><td>--</td><td>>0.65mmol/l
→ 24/kcal(kgBW/day
<0.65mmol/l
→ 6/kcal(kgBW/day
(+Phophate substitution)</td><td>Increase with 6 kcal/kgBW/d if phosphhate is >0.65 mmol/l until 24 kcal/kg/BW/day</td></tr>
</table>
Table is based on: Elke G, Hartl WH, Kreymann KG, Adolph M, Felbinger TW, Graf T, de Heer G, Heller AR, Kampa U, Mayer K, Muhl E, Niemann B, Rümelin A, Steiner S, Stoppe C, Weimann A, Bischoff SC. DGEM-Leitlinie: „Klinische Ernährung in der Intensivmedizin“. Aktuel Ernahrungsmed. 2018;43(05):341-408. DOI: 10.1055/a-0713-8179
</td></tr>
</table>

Main indicator VIII

Name of the indicator	**Structured communication with patients and relatives (next of kin)**
Dimension	Risk and effectiveness
Type of indicator	Process and outcome
Justification	Intensive care, including elective or emergency admissions, must be consistent with the patient’s will. The patient’s expectations and goals have to correspond with the treatment goals of intensive care therapy. In the course of ICU treatment, it is necessary to adjust planned and achieved medical and nursing therapy results to the patient’s will to avoid possible harm to the patient, his/her relatives, and also to caregivers.<br>To achieve this harmonization structured discussions between the treating physicians, nurses and therapists and the patient (and/or) with his/her relatives or authorized persons are necessary. The success of this communication depends on structure and technique of these discussions carried out by the intensive care physicians and nurses.
PICO	Structured communication and documentation of the discussion results leads to the prevention of stress disorders and depression in the patients’ relatives. Evidence-based communication techniques that focus on finding common therapy goals among discussion participants protect patients, family members, and staff from preventable stress.
Quality goal	Improvement of communication with patients and relatives and documentation of structured discussions. Avoidance of post-traumatic stress disorder (PTSD), depression, and anxiety in the patients’ family members. Avoidance of ethical and interpersonal conflicts among ICU staff.
Process quality numerator	First structured communication by a qualified physician within 72 hours after admission, including written documentation (standard value 98%) and one structured and documented communication per week
Denominator	All ICU patients after a stay of >72 hours
Outcome quality	Use of feedback platforms to evaluate patient and family satisfaction, e.g. family surveys, patient diaries (annual verification)

Explanation of terminology	Communication with patients and relatives in the ICU includes many forms. Consistent specification of therapeutic goals can only be achieved through structured discussions and their documentation (including date, participants, and content). The use of forms or templates in an electronic PDMS is recommended. In order to ensure continuity of care in case of treatment restrictions, appropriate templates should be included in the patient file.<br>Within 72 hours of admission to the ICU, an initial discussion should take place, followed by a follow-up interview at least once a week. The patient or his/her family/legal representative, a treating intensive care physician, a nurse, and optionally other disciplines involved in the treatment should take part in the discussions. This structured communication should be documented in a formal way. Contents should include:<br>1. Explanation of the current status of the patient<br>2. Determination of the patient's will, de facto or presumed, by the patient him-/herself or his/her representatives. Determination of the perspective of family members if the patient is unable to communicate<br>3. Current treatment options. Further treatment planning taking into account point 2 (→ treatment offer)<br>4. Treatment offer that aligns patient's will and therapy goals.<br>5. Medium- and long-term prognosis considering the probability of success of a therapy<br>6. Conclusion/determinations/consequences<br>The communication should balance different levels of information which exist between hospital staff and patients as well as their relatives. This supports the position of patients and relatives by empowering patient-side participants. The discussion should, therefore, follow current recommendations (i.e. VALUE concept). Additionally, the use of patient diaries to support family members is recommended. Feelings of guilt have to be avoided, and the patient's will has to be affirmed, ("what would he/she have thought and said if he/she would be sitting here now?").The aim is to achieve participatory decision-making (i.e. "shared decision-making"). The comprehensive information of patients or their relatives about different treatment options and their consequences should be seen as a prerequisite for this. Since there are always different approaches to treatment, the emotional, cultural or religious needs of patients and relatives have to be integrated into the decision-making-process.<br>ICU-diaries: An ICU-diary is an effective, evidence-based tool to prevent anxiety and depression after an ICU-stay for patients and their families. Daily entries help patients to close memory gaps after treatment. Missing or unreal memories are restored or corrected – the state of health or illness is visualized, memory gaps can be closed more effectively, and the entire situation of the ICU stay can be understood and processed better.<br>Family surveys or other forms of external feedback may help to identify communication deficiencies.
Source of data	Patient file, PDMS<br>Query: peer review
Standard value	98% appropriately documented communications
Level of evidence	Expert consensus
QUALIFY	Optional evaluation
AG-members	M. Brauchle, J.-P. Braun, A. Brinkmann, P. Czorlich, O. Kumpf, M. Ufelmann, R. Wildenauer
Conflicts of interest	See Attachment 2
Literature	[8, 46–55]
Additional information	**VALUE-concept:**<br>**V** Value family statements<br>**A** Acknowledge family emotions<br>**L** Listen to the family<br>**U** Understand the patient as a person<br>**E** Elicit family questions

	**Shared decision making (SDM):**<br>**S**eeking participation<br>**H**elping<br>**A**ssesing values und preferences<br>**R**eaching a decision<br>**E**valuating the decision<br>**Intensive care diaries:**<br>http://www.intensivtagebuch.de/<br>Relatives: anyone with whom patients have a significant relationship (e.g. family members, spouse/partner, friends)

Main indicator IX

Name of the indicator	**Early mobilisation**
Dimension	Risk and effectiveness
Type of indicator	Process and outcome
Justification	Early mobilization, i.e. mobilization within 72 hours after admission to intensive care, is an energy-consuming process aiming at maintaining or improving the regenerative capacity of muscles and thus the function, especially the mobility, of a critically ill patient.<br>The quality indicator includes:<br>1. Existence of an institutional standard<br>2. Implementation of this standard<br><br>Objectives of early mobilization are the improvement of pulmonary function, maintaining and improving muscular regeneration capacity and function, cardiovascular training, support of the weaning process, daily training, promotion of psychosocial well-being, reorientation and stimulation of vigilance and cognition with the aim of avoiding or shortening delirium, improved communication and initiation of early rehabilitation. Additionally objectives include avoidance of complications such as contractures, decubitus, pneumonia, etc.<br>The results are a significantly shorter respiration and length-of-stay in the ICU and increased functional independence upon discharge from the hospital. The effects seem best when a) patients are mobilized on a protocol basis; and b) critically ill patients with mild to moderate disease are treated; especially those with impaired consciousness benefit from early mobilization. The frequency, duration, and intensity of mobilization for specific conditions remains unclear.<br>Possible forms of early mobilization are:<br>• Passive: heart bed, bed mobility, neuromuscular electrical stimulation (NMES), robotics<br>• Assisted: bed bike, functional exercises, resistance exercises, transfers<br>• Active: active exercises, activities of daily living, walking<br>Early mobilization refers to the gradual mobilization of the critically ill patient within the first 72 hours after intensive care.<br>It is recommended to integrate measures for early mobilization into a treatment concept and to create a standardized algorithm for this, which is then implemented in a patient-adapted manner. In addition, it is recommended to order medically necessary immobilization always explicitly.
PICO	Do critically ill patients whose mobilization started within 72 hours of intensive care have a better functional outcome than critically ill patients who were not mobilized early and on a structured basis?
Quality goal	Ensuring sufficient nursing and physiotherapeutic resources for early mobilization
Process quality numerator	Number of patients with early mobilization
Denominator	Number of patients admitted to ICUs
Outcome quality numerator	Patient days without ordering non-mobilization without medical reason
Denominator	All patient days

Explanation of terminology	Various professions are involved in early mobilization, including specialist nurses and physiotherapists. Criteria for early mobilization should be defined on an institutional level. In this context, the advice published in a consensus recommendation describing the safety of mobilization measures in relation to applied invasive therapeutic procedures may be helpful.
Source of data	1. SOP/Standard available<br>2. Patient record, PDMS, care documentation<br>Query, peer review
Standard value	1. Structure: Presence of an algorithm for early mobilization<br>Standard or SOP/algorithm available? Yes/no<br>Yes=100%<br>2. Process: (Implementation) immobilization is ordered in writing; implementation yes/no; yes>90%<br>3. Total number of immobilized patients without indication=0
Level of evidence	Expert consensus
QUALIFY	Optional evaluation
AG-members	R. Dubb, A. Kaltwasser, S. J. Schaller, P. Nydahl
Conflicts of interest	See Attachment 2
Literature	[56–64]

Main indicator X

Name of the indicator	**Direction of the intensive care unit**
Dimension	Appropriateness, risk and effectiveness
Type of indicator	Structure
Justification	The management of the ICU by a certified intensivist who has no other clinical obligations, the presence of a certified intensivist during the core working period, and the presence of intensive care physicians and nursing staff over 24 h ensures the quality of the care and reduces mortality and treatment duration of ICU patients.<br>High-quality care of ICU patients requires the presence of experienced staff around the clock. Management level nurses and physicians as well as hospital administration have to ensure the implementation of the personnel requirements of the DIVI together with the hospital-management.
PICO	Not applicable
Quality goal	See justification above
Structure quality numerator	Number of days with fulfillment of structural specifications
Denominator	All days of the year over the observed period
Explanation of terminology	Personal presence of a certified intensivist in the core working time is considered necessary. In the literature, outcome-relevant structural specifications corresponding to the QI X can be found. The ICU has to be staffed with medical and nursing staff who is not assigned any other obligation and who is aware of the current problems of the patients.
Source of data	Personnel department, duty roster
Standard value	97% of days with compliance/year
Level of evidence	Expert consensus
QUALIFY	Optional evaluation
AG-members	J. Braun, A. Brinkmann, P. Czorlich, R. Dubb, A. Kaltwasser, O. Kumpf, A. Markewitz, G. Marx, E. Muhl, S. Pelz, R. Riessen, R. Wildenauer, G. Wöbker, H. Wrigge
Conflicts of interest	See Attachment 2
Literature	[65–73]
Additional information	At the time of publication, this indicator will be adapted according to the recently published recommendations of the DIVI for the structure of intensive care units.

References

1. von Dincklage F, Suchodolski K, Lichtner G, Friesdorf W, Podtschaske B, Ragaller M. Investigation of the usability of computerized critical care information systems in Germany. J Intensive Care Med. 2019 Mar;34(3):227-237. DOI: 10.1177/0885066617696848
2. Barcellos RA, Chatkin JM. Impact of a multidisciplinary checklist on the duration of invasive mechanical ventilation and length of ICU stay. J Bras Pneumol. 2020;46(3):e20180261. DOI: 10.36416/1806-3756/e20180261
3. Cifra CL, Houston M, Otto A, Kamath SS. Prompting rounding teams to address a daily best practice checklist in a pediatric intensive care unit. Jt Comm J Qual Patient Saf. 2019 Aug;45(8):543-51. DOI: 10.1016/j.jcjq.2019.05.012
4. Ervin JN, Kahn JM, Cohen TR, Weingart LR. Teamwork in the intensive care unit. Am Psychol. 2018;73(4):468-77. DOI: 10.1037/amp0000247
5. Hallam BD, Kuza CC, Rak K, Fleck JC, Heuston MM, Saha D, Kahn JM. Perceptions of rounding checklists in the intensive care unit: a qualitative study. BMJ Qual Saf. 2018 Oct;27(10):836-43. DOI: 10.1136/bmjqs-2017-007218
6. Lane D, Ferri M, Lemaire J, McLaughlin K, Stelfox HT. A systematic review of evidence-informed practices for patient care rounds in the ICU*. Crit Care Med. 2013 Aug;41(8):2015-29. DOI: 10.1097/CCM.0b013e31828a435f
7. Marra A, Ely EW, Pandharipande PP, Patel MB. The ABCDEF Bundle in Critical Care. Crit Care Clin. 2017 Apr;33(2):225-43. DOI: 10.1016/j.ccc.2016.12.005
8. Michalsen A, Neitzke G, Dutzmann J, Rogge A, Seidlein AH, Jöbges S, Burchardi H, Hartog C, Nauck F, Salomon F, Duttge G, Michels G, Knochel K, Meier S, Gretenkort P, Janssens U. Überversorgung in der Intensivmedizin: erkennen, benennen, vermeiden: Positionspapier der Sektion Ethik der DIVI und der Sektion Ethik der DGIIN [Overtreatment in intensive care medicine-recognition, designation, and avoidance: Position paper of the Ethics Section of the DIVI and the Ethics section of the DGIIN]. Med Klin Intensivmed Notfmed. 2021 May;116(4):281-94. DOI: 10.1007/s00063-021-00794-4
9. Pun BT, Balas MC, Barnes-Daly MA, Thompson JL, Aldrich JM, Barr J, Byrum D, Carson SS, Devlin JW, Engel HJ, Esbrook CL, Hargett KD, Harmon L, Hielsberg C, Jackson JC, Kelly TL, Kumar V, Millner L, Morse A, Perme CS, Posa PJ, Puntillo KA, Schweickert WD, Stollings JL, Tan A, D'Agostino McGowan L, Ely EW. Caring for critically ill patients with the ABCDEF Bundle: results of the ICU Liberation Collaborative in Over 15,000 Adults. Crit Care Med. 2019 Jan;47(1):3-14. DOI: 10.1097/CCM.0000000000003482
10. Riessen R, Celebi N, Weyrich P, Haap M. Die Visite auf der Intensivstation. German Interdisciplinary Journal of Intensive Care Medicine. 2011;48(6):403-10.
11. Cavalcanti AB, Bozza FA, Machado FR, Salluh JI, Campagnucci VP, Vendramim P, Guimaraes HP, Normilio-Silva K, Damiani LP, Romano E, Carrara F, Lubarino Diniz de Souza J, Silva AR, Ramos GV, Teixeira C, Brandão da Silva N, Chang CC, Angus DC, Berwanger O; Writing Group for the CHECKLIST-ICU Investigators and the Brazilian Research in Intensive Care Network (BRICNet). Effect of a quality improvement intervention with daily round checklists, goal setting, and clinician prompting on mortality of critically ill patients: a randomized clinical trial. JAMA. 2016 Apr;315(14):1480-90. DOI: 10.1001/jama.2016.3463
12. AWMF. Analgesie, Sedierung und Delirmanagement in der Intensivmedizin (DAS-Leitlinie)[Internet]. 2021 [updated 31.03.2021]. Verfügbar unter: https://www.awmf.org/uploads/tx_szleitlinien/001-012l_S3_Analgesie-Sedierung-Delirmanagement-in-der-Intensivmedizin-DAS_2021-08.pdf
13. Bellani G, Laffey JG, Pham T, Fan E, Brochard L, Esteban A, Gattinoni L, van Haren F, Larsson A, McAuley DF, Ranieri M, Rubenfeld G, Thompson BT, Wrigge H, Slutsky AS, Pesenti A; LUNG SAFE Investigators; ESICM Trials Group. Epidemiology, patterns of care, and mortality for patients with Acute Respiratory Distress Syndrome in intensive care units in 50 Countries. JAMA. 2016 Feb;315(8):788-800. DOI: 10.1001/jama.2016.0291
14. Amato MB, Meade MO, Slutsky AS, Brochard L, Costa EL, Schoenfeld DA, Stewart TE, Briel M, Talmor D, Mercat A, Richard JC, Carvalho CR, Brower RG. Driving pressure and survival in the acute respiratory distress syndrome. N Engl J Med. 2015 Feb;372(8):747-55. DOI: 10.1056/NEJMsa1410639
15. AWMF. Invasive Beatmung und Einsatz extrakorporaler Verfahren bei akuter respiratorischer Insuffizienz [Internet].2017 [Stand: 20.11.2017 (in Überarbeitung)]. Verfügbar unter: http://www.awmf.org/leitlinien/detail/ll/001-021.html
16. Bein T, Bischoff M, Brückner U, Gebhardt K, Henzler D, Hermes C, Lewandowski K, Max M, Nothacker M, Staudinger T, Tryba M, Weber-Carstens S, Wrigge H; Deutsche Gesellschaft für Anästhesiologie und Intensivmedizin. Kurzversion S2e-Leitlinie - „Lagerungstherapie und Frühmobilisation zur Prophylaxe oder Therapie von pulmonalen Funktionsstörungen“ [Short version S2e guidelines: “Positioning therapy and early mobilization for prophylaxis or therapy of pulmonary function disorders”]. Anaesthesist. 2015 Aug;64(8):596-611. DOI: 10.1007/s00101-015-0060-4
17. Brower RG, Lanken PN, MacIntyre N, Matthay MA, Morris A, Ancukiewicz M, Schoenfeld D, Thompson BT; National Heart, Lung, and Blood Institute ARDS Clinical Trials Network. Higher versus lower positive end-expiratory pressures in patients with the acute respiratory distress syndrome. N Engl J Med. 2004 Jul;351(4):327-36. DOI: 10.1056/NEJMoa032193
18. Combes A, Peek GJ, Hajage D, Hardy P, Abrams D, Schmidt M, Dechartres A, Elbourne D. ECMO for severe ARDS: systematic review and individual patient data meta-analysis. Intensive Care Med. 2020 Nov;46(11):2048-57. DOI: 10.1007/s00134-020-06248-3

19. Guérin C, Reignier J, Richard JC, Beuret P, Gacouin A, Boulain T, Mercier E, Badet M, Mercat A, Baudin O, Clavel M, Chatellier D, Jaber S, Rosselli S, Mancebo J, Sirodot M, Hilbert G, Bengler C, Richecoeur J, Gainnier M, Bayle F, Bourdin G, Leray V, Girard R, Baboi L, Ayzac L; PROSEVA Study Group. Prone positioning in severe acute respiratory distress syndrome. N Engl J Med. 2013 Jun;368(23):2159-68. DOI: 10.1056/NEJMoa1214103

20. Brower RG, Matthay MA, Morris A, Schoenfeld D, Thompson BT, Wheeler A; Acute Respiratory Distress Syndrome Network. Ventilation with lower tidal volumes as compared with traditional tidal volumes for acute lung injury and the acute respiratory distress syndrome. N Engl J Med. 2000 May 4;342(18):1301-8. DOI: 10.1056/NEJM200005043421801

21. Ranieri VM, Rubenfeld GD, Thompson BT, Ferguson ND, Caldwell E, Fan E, Camporota L, Slutsky AS; ARDS Definition Task Force. Acute respiratory distress syndrome: the Berlin Definition. JAMA. 2012 Jun 20;307(23):2526-33. DOI: 10.1001/jama.2012.5669

22. Ouellette DR, Patel S, Girard TD, Morris PE, Schmidt GA, Truwit JD, Alhazzani W, Burns SM, Epstein SK, Esteban A, Fan E, Ferrer M, Fraser GL, Gong MN, Hough CL, Mehta S, Nanchal R, Pawlik AJ, Schweickert WD, Sessler CN, Strøm T, Kress JP. Liberation from mechanical ventilation in critically ill adults: an Official American College of Chest Physicians/American Thoracic Society Clinical Practice Guideline: Inspiratory pressure augmentation during spontaneous breathing trials, protocols minimizing sedation, and noninvasive ventilation immediately after extubation. Chest. 2017 Jan;151(1):166-80. DOI: 10.1016/j.chest.2016.10.036

23. Schönhofer B, Geiseler J, Dellweg D, Fuchs H, Moerer O, Weber-Carstens S, Westhoff M, Windisch W, Hirschfeld-Araujo J, Janssens U, Rollnik J, Rosseau S, Schreiter D, Sitter H; Weitere beteiligte wissenschaftliche Fachgesellschaften und Institutionen: Deutsche Gesellschaft für Anästhesiologie und Intensivmedizin e. V. (DGAI); Deutsche Gesellschaft für Chirurgie e. V. (DGCH); Deutsche Gesellschaft für Ernährungsmedizin e. V. (DGEM); Deutsche Gesellschaft für Geriatrie e. V. (DGG); Deutsche Gesellschaft für Internistische Intensivmedizin und Notfallmedizin e. V. (DGIIN); Deutsche Gesellschaft für Kardiologie – Herz- und Kreislaufforschung e. V. (DGK); Deutsche Gesellschaft für Neurointensiv- und Notfallmedizin e. V. (DGNI); Deutsche Gesellschaft für Neurorehabilitation e. V. (DGNR); Deutsche Gesellschaft für Palliativmedizin e. V. (DGP); Deutsche Interdisziplinäre Vereinigung für Intensiv- und Notfallmedizin e. V. (DIVI)); Gesellschaft für Neonatologie und pädiatrische Intensivmedizin e. V. (GNPI); Deutsche Gesellschaft für Neurochirurgie e. V. (DGNC); Deutsche Gesellschaft für Neurologie e. V. (DGN); Deutschsprachige Medizinische Gesellschaft für Paraplegie e. V. (DMPG); Deutsche Gesellschaft für Thorax-, Herz- und Gefäßchirurgie e. V. (DGTHG); Deutsche Gesellschaft für Fachkrankenpflege und Funktionsdienste e. V. (DGF); Deutsche Interdisziplinäre Gesellschaft für Außerklinische Beatmung e. V. (DIGAB); Deutscher Verband für Physiotherapie e. V. (ZVK); Deutscher Bundesverband für Logopädie e. V. (dbl). Prolongiertes Weaning [Prolonged Weaning – S2k-Guideline published by the German Respiratory Society]. Pneumologie. 2019 Dec;73(12):723-814. DOI: 10.1055/a-1010-8764

24. Kommission für Krankenhaushygiene und Infektionsprävention, Robert Koch Institute (KRINKO). Anforderungen an die Hygiene bei Punktionen und Injektionen [Public health requirements in punctures and injections]. Bundesgesundheitsblatt Gesundheitsforschung Gesundheitsschutz. 2011 Sep;54(9):1135-44. DOI: 10.1007/s00103-011-1352-8

25. Kommission für Krankenhaushygiene und Infektionsprävention, Robert Koch Institute (KRINKO).Prävention der nosokomialen beatmungsassoziierten Pneumonie. Empfehlung der Kommission für Krankenhaushygiene und Infektionsprävention (KRINKO) beim Robert Koch-Institut [Prevention of nosocomial ventilator-associated pneumonia. The Commission for Hospital Hygiene and Infection Prevention (KRINKO) at the Robert Koch Institute]. Bundesgesundheitsblatt Gesundheitsforschung Gesundheitsschutz. 2013 Nov;56(11):1578-90.

26. Kommission für Krankenhaushygiene und Infektionsprävention, Robert Koch Institute (KRINKO). Prävention und Kontrolle Katheter-assoziierter Harnwegsinfektionen: Empfehlung der Kommission für Krankenhaushygiene und Infektionsprävention (KRINKO) beim Robert Koch-Institut. Bundesgesundheitsblatt Gesundheitsforschung Gesundheitsschutz. 2015 Jun;58(6):641-50. DOI: 10.1007/s00103-015-2152-3

27. Kommission für Krankenhaushygiene und Infektionsprävention, Robert Koch Institute (KRINKO). Prävention von Infektionen, die von Gefäßkathetern ausgehen: Teil 1 – Nichtgetunnelte zentralvenöse Katheter. Empfehlung der Kommission für Krankenhaushygiene und Infektionsprävention (KRINKO) beim Robert Koch-Institut. Bundesgesundheitsblatt Gesundheitsforschung Gesundheitsschutz. 2017 Feb;60(2):171-206. DOI: 10.1007/s00103-016-2487-4

28. Zhao T, Wu X, Zhang Q, Li C, Worthington HV, Hua F. Oral hygiene care for critically ill patients to prevent ventilator-associated pneumonia. Cochrane Database Syst Rev. 2020 Dec;12(12):CD008367. DOI: 10.1002/14651858.CD008367.pub4

29. Brunkhorst FM, Weigand MA, Pletz M, Gastmeier P, Lemmen SW, Meier-Hellmann A, Ragaller M, Weyland A, Marx G, Bucher M, Gerlach H, Salzberger B, Grabein B, Welte T, Werdan K, Kluge S, Bone HG, Putensen C, Rossaint R, Quintel M, Spies C, Weiß B, John S, Oppert M, Jörres A, Brenner T, Elke G, Gründling M, Mayer K, Weimann A, Felbinger TW, Axer H; Deutsche Sepsis Gesellschaft e. V. S3-Leitlinie Sepsis – Prävention, Diagnose, Therapie und Nachsorge: Langfassung [S3 Guideline Sepsis-prevention, diagnosis, therapy, and aftercare: Long version]. Med Klin Intensivmed Notfmed. 2020 May;115(Suppl 2):37-109. DOI: 10.1007/s00063-020-00685-0

30. Evans L, Rhodes A, Alhazzani W, Antonelli M, Coopersmith CM, French C, Machado FR, Mcintyre L, Ostermann M, Prescott HC, Schorr C, Simpson S, Wiersinga WJ, Alshamsi F, Angus DC, Arabi Y, Azevedo L, Beale R, Beilman G, Belley-Cote E, Burry L, Cecconi M, Centofanti J, Coz Yataco A, De Waele J, Dellinger RP, Doi K, Du B, Estenssoro E, Ferrer R, Gomersall C, Hodgson C, Hylander Møller M, Iwashyna T, Jacob S, Kleinpell R, Klompas M, Koh Y, Kumar A, Kwizera A, Lobo S, Masur H, McGloughlin S, Mehta S, Mehta Y, Mer M, Nunnally M, Oczkowski S, Osborn T, Papathanassoglou E, Perner A, Puskarich M, Roberts J, Schweickert W, Seckel M, Sevransky J, Sprung CL, Welte

T, Zimmerman J, Levy M. Surviving Sepsis Campaign: International guidelines for management of sepsis and septic shock 2021. Crit Care Med. 2021 Nov 1;49(11):e1063-e1143. DOI: 10.1097/CCM.0000000000005337

31. Kommission für Krankenhaushygiene und Infektionsprävention, Robert Koch Institute (KRINKO). Prävention von Infektionen, die von Gefäßkathetern ausgehen: Hinweise zur Implementierung Informativer Anhang 2 zur Empfehlung der Kommission für Krankenhaushygiene und Infektionsprävention (KRINKO) beim Robert Koch-Institut. Bundesgesundheitsblatt Gesundheitsforschung Gesundheitsschutz. 2017 Feb;60(2):231-44. DOI: 10.1007/s00103-016-2486-5
32. Karch A, Castell S, Schwab F, Geffers C, Bongartz H, Brunkhorst FM, Gastmeier P, Mikolajczyk RT. Proposing an empirically justified reference threshold for blood culture sampling rates in intensive care units. J Clin Microbiol. 2015 Feb;53(2):648-52. DOI: 10.1128/JCM.02944-14
33. Brinkmann A, Röhr AC, Frey OR, Krüger WA, Brenner T, Richter DC, Bodmann KF, Kresken M, Grabein B. S2k-Leitlinie der PEG zur kalkulierten parenteralen Initialtherapie bakterieller Erkrankungen bei Erwachsenen: Fokussierte Zusammenfassung und ergänzende Informationen zur Antibiotikatherapie kritisch kranker Patienten [S2k guidelines of the PEG on calculated parenteral initial treatment of bacterial diseases in adults: Focussed summary and supplementary information on antibiotic treatment of critically ill patients]. Anaesthesist. 2018 Dec;67(12):936-49. DOI: 10.1007/s00101-018-0512-8
34. Röhr AC, Köberer A, Fuchs T, von Freyberg P, Frey OR, Brinkmann A. SOP Individuelle Dosierung und Applikation von Antiinfektiva auf der Intensivstation. Intensivmedizin up2date. 2018;14(03):238-43. DOI: 10.1055/a-0626-8184
35. Bloos F, Rüddel H, Thomas-Rüddel D, Schwarzkopf D, Pausch C, Harbarth S, Schreiber T, Gründling M, Marshall J, Simon P, Levy MM, Weiss M, Weyland A, Gerlach H, Schürholz T, Engel C, Matthäus-Krämer C, Scheer C, Bach F, Riessen R, Poidinger B, Dey K, Weiler N, Meier-Hellmann A, Häberle HH, Wöbker G, Kaisers UX, Reinhart K; MEDUSA study group. Effect of a multifaceted educational intervention for anti-infectious measures on sepsis mortality: a cluster randomized trial. Intensive Care Med. 2017 Nov;43(11):1602-12. DOI: 10.1007/s00134-017-4782-4
36. Leone M, Pulcini C, De Waele J. Improving care for the ICU patient with suspected infection: a multidisciplinary perspective. Clin Microbiol Infect. 2020 Jan;26(1):6-7. DOI: 10.1016/j.cmi.2019.07.020
37. Abdul-Aziz MH, Alffenaar JC, Bassetti M, Bracht H, Dimopoulos G, Marriott D, Neely MN, Paiva JA, Pea F, Sjovall F, Timsit JF, Udy AA, Wicha SG, Zeitlinger M, De Waele JJ, Roberts JA; Infection Section of European Society of Intensive Care Medicine (ESICM); Pharmacokinetic/pharmacodynamic and Critically Ill Patient Study Groups of European Society of Clinical Microbiology and Infectious Diseases (ESCMID); Infectious Diseases Group of International Association of Therapeutic Drug Monitoring and Clinical Toxicology (IATDMCT); Infections in the ICU and Sepsis Working Group of International Society of Antimicrobial Chemotherapy (ISAC). Antimicrobial therapeutic drug monitoring in critically ill adult patients: a position paper. Intensive Care Med. 2020 Jun;46(6):1127-53. DOI: 10.1007/s00134-020-06050-1
38. Schuts EC, Hulscher MEJL, Mouton JW, Verduin CM, Stuart JWTC, Overdiek HWPM, van der Linden PD, Natsch S, Hertogh CMPM, Wolfs TFW, Schouten JA, Kullberg BJ, Prins JM. Current evidence on hospital antimicrobial stewardship objectives: a systematic review and meta-analysis. Lancet Infect Dis. 2016 Jul;16(7):847-56. DOI: 10.1016/S1473-3099(16)00065-7
39. Derendorf H, Heinrichs T, Reimers T, Lebert C, Brinkmann A. Calculated initial parenteral treatment of bacterial infections: pharmacokinetics and pharmacodynamics. GMS Infect Dis. 2020 Mar 26;8:Doc17. DOI: 10.3205/id000061
40. Bassetti M, Poulakou G, Ruppe E, Bouza E, Van Hal SJ, Brink A. Antimicrobial resistance in the next 30 years, humankind, bugs and drugs: a visionary approach. Intensive Care Med. 2017 Oct;43(10):1464-75. DOI: 10.1007/s00134-017-4878-x
41. Brunkhorst FM, Weigand MA, Pletz M, Gastmeier P, Lemmen SW, Meier-Hellmann A, Ragaller M, Weyland A, Marx G, Bucher M, Gerlach H, Salzberger B, Grabein B, Welte T, Werdan K, Kluge S, Bone HG, Putensen C, Rossaint R, Quintel M, Spies C, Weiß B, John S, Oppert M, Jörres A, Brenner T, Elke G, Gründling M, Mayer K, Weimann A, Felbinger TW, Axer H, Heller T, Gagelmann N; Deutsche Sepsis Gesellschaft e. V. S3-Leitlinie Sepsis – Prävention, Diagnose, Therapie und Nachsorge: Zusammenfassung starker Empfehlungen [S3 guideline sepsis-prevention, diagnosis, treatment, and aftercare: summary of the strong recommendations]. Med Klin Intensivmed Notfmed. 2020 Apr;115(3):178-88. DOI: 10.1007/s00063-020-00671-6
42. Cusumano JA, Klinker KP, Huttner A, Luther MK, Roberts JA, LaPlante KL. Towards precision medicine: Therapeutic drug monitoring-guided dosing of vancomycin and β-lactam antibiotics to maximize effectiveness and minimize toxicity. Am J Health Syst Pharm. 2020 Jul;77(14):1104-12. DOI: 10.1093/ajhp/zxaa128
43. Tabah A, Bassetti M, Kollef MH, Zahar JR, Paiva JA, Timsit JF, Roberts JA, Schouten J, Giamarellou H, Rello J, De Waele J, Shorr AF, Leone M, Poulakou G, Depuydt P, Garnacho-Montero J. Antimicrobial de-escalation in critically ill patients: a position statement from a task force of the European Society of Intensive Care Medicine (ESICM) and European Society of Clinical Microbiology and Infectious Diseases (ESCMID) Critically Ill Patients Study Group (ESGCIP). Intensive Care Med. 2020 Feb;46(2):245-65. DOI: 10.1007/s00134-019-05866-w
44. Elke G, Hartl WH, Kreymann KG, Adolph M, Felbinger TW, Graf T, de Heer G, Heller AR, Kampa U, Mayer K, Muhl E, Niemann B, Rümelin A, Steiner S, Stoppe C, Weimann A, Bischoff SC. DGEM-Leitlinie: „Klinische Ernährung in der Intensivmedizin". Aktuel Ernahrungsmed. 2018;43(05):341-408. DOI: 10.1055/a-0713-8179
45. DNQP. Expertenstandard „Ernährungsmanagement zur Sicherung und Förderung der oralen Ernährung in der Pflege – 1. Aktualisierung 2017". Osnabrück: Deutsches Netzwerk für Qualitätsentwicklung in der Pflege; 2017.

46. Michalsen A, Long AC, DeKeyser Ganz F, White DB, Jensen HI, Metaxa V, Hartog CS, Latour JM, Truog RD, Kesecioglu J, Mahn AR, Curtis JR. Interprofessional shared decision-making in the ICU: a systematic review and recommendations from an expert panel. Crit Care Med. 2019 Sep;47(9):1258-66. DOI: 10.1097/CCM.0000000000003870
47. Kumpf O, Ostmeier S, Braun JP, Spies C, Haase U, Denke C, Jöbges S. Wie sollte man ein strukturiertes Angehörigengespräch auf einer Intensivstation führen und dokumentieren? Anästh Intensivmed. 2019;60(5):244–53. DOI: 10.19224/ai2019.244
48. Kerckhoffs MC, Senekal J, van Dijk D, Artigas A, Butler J, Michalsen A, van Mol MMC, Moreno R, Pais da Silva F, Picetti E, Póvoa P, Robertsen A, van Delden JJM. Framework to support the process of decision-making on life-sustaining treatments in the ICU: results of a Delphi Study. Crit Care Med. 2020 May;48(5):645-53. DOI: 10.1097/CCM.0000000000004221
49. Ullman AJ, Aitken LM, Rattray J, Kenardy J, Le Brocque R, MacGillivray S, Hull AM. Intensive care diaries to promote recovery for patients and families after critical illness: a Cochrane Systematic Review. Int J Nurs Stud. 2015 Jul;52(7):1243-53. DOI: 10.1016/j.ijnurstu.2015.03.020
50. Curtis JR, White DB. Practical guidance for evidence-based ICU family conferences. Chest. 2008 Oct;134(4):835-43. DOI: 10.1378/chest.08-0235
51. Hartog CS, Jöbges S, Kumpf O, Janssens U. Das Angehörigengespräch in der Intensivmedizin: Grundlagen und Empfehlungen für die Praxis [Communicating with families in the ICU: background and practical recommendations]. Med Klin Intensivmed Notfmed. 2018 Apr;113(3):231-42. DOI: 10.1007/s00063-018-0417-y
52. Jones C, Bäckman C, Capuzzo M, Egerod I, Flaatten H, Granja C, Rylander C, Griffiths RD; RACHEL group. Intensive care diaries reduce new onset post traumatic stress disorder following critical illness: a randomised, controlled trial. Crit Care. 2010;14(5):R168. DOI: 10.1186/cc9260
53. Long AC, Brumback LC, Curtis JR, Avidan A, Baras M, De Robertis E, Efferen L, Engelberg RA, Kross EK, Michalsen A, Mularski RA, Sprung CL; Worldwide End-of-Life Practice for Patients in ICUs (WELPICUS) Investigators. Agreement with consensus statements on end-of-life care: a description of variability at the level of the provider, hospital, and country. Crit Care Med. 2019 Oct;47(10):1396-401. DOI: 10.1097/CCM.0000000000003922
54. Nydahl P, Fischill M, Deffner T, Neudeck V, Heindl P. Intensivtagebücher senken Risiko für psychische Folgestörungen: Systematische Literaturrecherche und Metaanalyse [Diaries for intensive care unit patients reduce the risk for psychological sequelae: systematic literature review and meta-analysis]. Med Klin Intensivmed Notfmed. 2019 Feb;114(1):68-76. DOI: 10.1007/s00063-018-0456-4
55. Parker AM, Sricharoenchai T, Raparla S, Schneck KW, Bienvenu OJ, Needham DM. Posttraumatic stress disorder in critical illness survivors: a metaanalysis. Crit Care Med. 2015 May;43(5):1121-9. DOI: 10.1097/CCM.0000000000000882
56. Ding N, Zhang Z, Zhang C, Yao L, Yang L, Jiang B, Wu Y, Jiang L, Tian J. What is the optimum time for initiation of early mobilization in mechanically ventilated patients? A network meta-analysis. PLoS One. 2019;14(10):e0223151. DOI: 10.1371/journal.pone.0223151
57. Waldauf P, Jiroutková K, Krajčová A, Puthucheary Z, Duška F. Effects of rehabilitation interventions on clinical outcomes in critically ill patients: systematic review and meta-analysis of randomized controlled trials. Crit Care Med. 2020 Jul;48(7):1055-65. DOI: 10.1097/CCM.0000000000004382
58. Schaller SJ, Anstey M, Blobner M, Edrich T, Grabitz SD, Gradwohl-Matis I, Heim M, Houle T, Kurth T, Latronico N, Lee J, Meyer MJ, Peponis T, Talmor D, Velmahos GC, Waak K, Walz JM, Zafonte R, Eikermann M; International Early SOMS-guided Mobilization Research Initiative. Early, goal-directed mobilisation in the surgical intensive care unit: a randomised controlled trial. Lancet. 2016 Oct;388(10052):1377-88. DOI: 10.1016/S0140-6736(16)31637-3
59. Bein T, Bischoff M, Brückner U, Gebhardt K, Henzler D, Hermes C, Lewandowski K, Max M, Nothacker M, Staudinger T, Tryba M, Weber-Carstens S, Wrigge H. S2e guideline: positioning and early mobilisation in prophylaxis or therapy of pulmonary disorders: Revision 2015: S2e guideline of the German Society of Anaesthesiology and Intensive Care Medicine (DGAI). Anaesthesist. 2015 Dec;64 Suppl 1:1-26. DOI: 10.1007/s00101-015-0071-1
60. Dubb R, Nydahl P, Hermes C, Schwabbauer N, Toonstra A, Parker AM, Kaltwasser A, Needham DM. Barriers and strategies for early mobilization of patients in intensive care units. Ann Am Thorac Soc. 2016 May;13(5):724-30. DOI: 10.1513/AnnalsATS.201509-586CME
61. Nydahl P, Dubb R, Filipovic S, Hermes C, Jüttner F, Kaltwasser A, Klarmann S, Mende H, Nessizius S, Rottensteiner C. Algorithmen zur Frühmobilisierung auf Intensivstationen [Algorithms for early mobilization in intensive care units]. Med Klin Intensivmed Notfmed. 2017 Mar;112(2):156-62. DOI: 10.1007/s00063-016-0210-8
62. Nydahl P, Sricharoenchai T, Chandra S, Kundt FS, Huang M, Fischill M, Needham DM. Safety of patient mobilization and rehabilitation in the intensive care unit. Systematic review with meta-analysis. Ann Am Thorac Soc. 2017 May;14(5):766-77. DOI: 10.1513/AnnalsATS.201611-843SR
63. Schaller SJ, Scheffenbichler FT, Bose S, Mazwi N, Deng H, Krebs F, Seifert CL, Kasotakis G, Grabitz SD, Latronico N, Houle T, Blobner M, Eikermann M. Influence of the initial level of consciousness on early, goal-directed mobilization: a post hoc analysis. Intensive Care Med. 2019 Feb;45(2):201-10. DOI: 10.1007/s00134-019-05528-x

64. Zhang L, Hu W, Cai Z, Liu J, Wu J, Deng Y, Yu K, Chen X, Zhu L, Ma J, Qin Y. Early mobilization of critically ill patients in the intensive care unit: a systematic review and meta-analysis. PLoS One. 2019;14(10):e0223185. DOI: 10.1371/journal.pone.0223185

65. Wallace DJ, Angus DC, Barnato AE, Kramer AA, Kahn JM. Nighttime intensivist staffing and mortality among critically ill patients. N Engl J Med. 2012 May;366(22):2093-101. DOI: 10.1056/NEJMsa1201918

66. Kerlin MP, Adhikari NK, Rose L, Wilcox ME, Bellamy CJ, Costa DK, Gershengorn HB, Halpern SD, Kahn JM, Lane-Fall MB, Wallace DJ, Weiss CH, Wunsch H, Cooke CR; ATS Ad Hoc Committee on ICU Organization. An Official American Thoracic Society Systematic Review: the effect of nighttime intensivist staffing on mortality and length of stay among intensive care unit patients. Am J Respir Crit Care Med. 2017 Feb;195(3):383-93. DOI: 10.1164/rccm.201611-2250ST

67. Jorch G, Kluge S, König F, Markewitz A, Notz K, Parvu V, Quintel M, Schneider D, Sybrecht GW, Waydhas C. Empfehlungen zur Struktur und Ausstattung von Intensivstationen [Internet]. 2010 [updated 30.11.2010]. Verfügbar unter: https://www.divi.de/joomlatools-files/docman-files/publikationen/intensivmedizin/20101130-publikationen-empfehlungen-zur-struktur-v-intensivstationen-langversion.pdf

68. Pronovost PJ, Angus DC, Dorman T, Robinson KA, Dremsizov TT, Young TL. Physician staffing patterns and clinical outcomes in critically ill patients: a systematic review. JAMA. 2002 Nov;288(17):2151-62. DOI: 10.1001/jama.288.17.2151

69. Pronovost PJ, Dang D, Dorman T, Lipsett PA, Garrett E, Jenckes M, Bass EB. Intensive care unit nurse staffing and the risk for complications after abdominal aortic surgery. Eff Clin Pract. 2001 Sep-Oct;4(5):199-206.

70. Pronovost PJ, Jenckes MW, Dorman T, Garrett E, Breslow MJ, Rosenfeld BA, Lipsett PA, Bass E. Organizational characteristics of intensive care units related to outcomes of abdominal aortic surgery. JAMA. 1999 Apr;281(14):1310-7. DOI: 10.1001/jama.281.14.1310

71. Rothen HU, Stricker K, Einfalt J, Bauer P, Metnitz PG, Moreno RP, Takala J. Variability in outcome and resource use in intensive care units. Intensive Care Med. 2007 Aug;33(8):1329-36. DOI: 10.1007/s00134-007-0690-3

72. Treggiari MM, Martin DP, Yanez ND, Caldwell E, Hudson LD, Rubenfeld GD. Effect of intensive care unit organizational model and structure on outcomes in patients with acute lung injury. Am J Respir Crit Care Med. 2007 Oct;176(7):685-90. DOI: 10.1164/rccm.200701-165OC

73. Vincent JL. Need for intensivists in intensive-care units. Lancet. 2000 Aug;356(9231):695-6. DOI: 10.1016/S0140-6736(00)02622-2
