# Supplementary material for: Quality indicators in intensive care medicine for Germany – fourth edition 2022
Source: Ger Med Sci. 2023 Jun 23;21:Doc10. doi: 10.3205/000324 (PMC10326525; doi:10.3205/000324)
Supplement: Potential conflicts of interest [file GMS-21-10-s-002.pdf]

Attachment 2

Potential conflicts of interest of the authors

Name	Function	Potential conflicts of interest
M. Assenheimer	Co-author	None declared
F. Bloos	Member NSPR	Received honorary for an expert board meeting by Baxter and an unrestricted non-financial support for a clinical trial by Associates of Cape Cod, Inc.
M. Brauchle	Co-author	None declared
J.-P. Braun	Member NSPR	None declared
A. Brinkmann	Member NSPR	Lecture fees and/or refunding of travel expenses from: Pfizer Pharma GmbH, Fresenius Medical Care (FMC), MSD Sharp & Dohme GmbH, Laboratory Volkmann, Karlsruhe, Labor Limbach
P. Czorlich	Member NSPR	Received consultation fees and holds stocks in small amounts from BioNTech SE and Curevac N.V.
C. Dame	Member NSPR	None declared
R. Dubb	Member NSPR	Lecture fees and refunding of travel expenses from Avanos. Refunding of travel expenses for lectures at national and international meetings and conferences. Member of DIVI, DGF, Netzwerk Frühmobilisation, and Deutsches Delirnetzwerk
G. Gahn	Member NSPR	None declared
C. A. Greim	Mitglied NSPR	None declared
B. Gruber	Co-author	None declared
H. Habermehl	Co-author	Received honorary from Getinge: Webinar “Forum für Atmungstherapie und Intensivbeatmung”, and from Liberate Medical LLC (Crestwood USA)
E. Herting	Member NSPR	None declared
A. Kaltwasser	Member NSPR	Lecture fees and/or refunding of travel expenses from Orion Pharma, Avanos. Gutachtertätigkeit für NOVO. Member of DIVI, DGF, DBfK, “Netzwerk Frühmobilisation”, and the German “Delirnetzwerk”
S. Krotsetis	Co-author	None declared
B. Kruger	Co-author	None declared
O. Kumpf	Speaker NSPR	Received consultation fees from INQUAM e.V. and holds shares from healthcare-related investment funds
A. Markewitz	Member NSPR	None declared
G. Marx	Member NSPR	None declared
E. Muhl	Member NSPR	None declared
P. Nydahl	Co-author	None declared
S. Pelz	Co-author	Membership in DGF (Germany), EfCCNa (Europe), DN APN & ANP (Germany)
R. Riessen	Member NSPR	Received honoraries and travel expenses from medical publishers (Thieme, Springer), Deutsche Stiftung Organtransplantation (DSO) and Vitalaire. Received travel expenses for talks at national and international meetings
M. Sasse	Member NSPR	None declared
S. J. Schaller	Co-author	Reports grants and non-financial support from ESICM (Brussels, Belgium), Fresenius (Germany), Liberate Medical LLC (Crestwood, USA), Reactive Robotics (Munich, Germany), STIMIT AG (Nidau, Switzerland) as well as from the Technical University of Munich, Germany, from national (e.g. DGAI) and international (e.g. ESICM) medical societies (or their congress organizers) in the field of anesthesiology and intensive care, personal fees and non-financial support from Bavarian Medical Association, all outside the submitted work. Holds stocks in small amounts from Alphabeth Inc., Rhön-Klinikum AG, and Siemens AG

A. Schäfer	Co-author	None declared
T. Schürholz	Member NSPR	None declared
M. Ufelmann	Co-author	None declared
C. Waydhas	Member NSPR	None declared
J. Weimann	Co-author	None declared
R. Wildenauer	Member NSPR	Holds shares from healthcare-related investment funds
G. Wöbker	Member NSPR	None declared
H. Wrigge	Member NSPR	Receives research support from Dompé, Milan, Italy, lecture fees from von Getinge, Rastatt, Germany, lectury fees from MSD, Konstanz, Germany, lecture fees from Arjo, Mainz-Kastel, Germany, and consulting fees from Liberate Medical, KY, USA

NSPR=Nationale Steuerungsgruppe Peer Review
